# Supplementary material for: Implementation of the Dutch expertise centre for child abuse: descriptive data from the first 4 years
Source: BMJ Open. 2019 Aug 21;9(8):e031008. doi: 10.1136/bmjopen-2019-031008 (PMC6707647; doi:10.1136/bmjopen-2019-031008)
Supplement: Author's manuscript [file bmjopen-2019-031008.draft_revisions.pdf]

BMJ Open

BMJ Open is committed to open peer review. As part of this commitment we make the peer review history of every article we publish publicly available.

When an article is published we post the peer reviewers' comments and the authors' responses online. We also post the versions of the paper that were used during peer review. These are the versions that the peer review comments apply to.

The versions of the paper that follow are the versions that were submitted during the peer review process. They are not the versions of record or the final published versions. They should not be cited or distributed as the published version of this manuscript.

BMJ Open is an open access journal and the full, final, typeset and author-corrected version of record of the manuscript is available on our site with no access controls, subscription charges or pay-per-view fees (http://bmjopen.bmj.com).

If you have any questions on BMJ Open's open peer review process please email info.bmjopen@bmj.com

BMJ Open

The start of the Dutch Expertise Centre for Child Abuse; a descriptive study of the implementation and data of the first 4 years

Journal:	*BMJ Open*
Manuscript ID	bmjopen-2019-031008
Article Type:	Research
Date Submitted by the Author:	11-Apr-2019
Complete List of Authors:	van Rijn, Rick; Emma Children's hospital, Academic Medical Center, radiology; Nederlands Forensisch Instituut, Forensic Medicine<br>Affourtit, Marjo; Erasmus MC Sophia Children's Hospital, Pediatrics<br>Karst, Wouter; Nederlands Forensisch Instituut, Forensic Medicine<br>Kamphuis, Mascha; Dutch Expertise Center for Child Abuse<br>de Bock, Leonie; Dutch Expertise Center for Child Abuse<br>van de Putte, Elise; University Medical Center Utrecht, General Paediatrics
Keywords:	Child protection < PAEDIATRICS, Child Abuse, FORENSIC MEDICINE

SCHOLARONE™
Manuscripts

The start of the Dutch Expertise Centre for Child Abuse; a descriptive study of the implementation and data of the first 4 years

R.R. van Rijn[1,2#,3], M.J. Affourtit[3,4], W.A. Karst[2,3], M. Kamphuis[3], L.C. de Bock[3], E. van de Putte[3,5], on behalf of the Dutch Expertise Centre for Child Abuse Study Group

Department of Radiology[1], Emma Children's Hospital - Amsterdam UMC, University of Amsterdam, Amsterdam, the Netherlands

Department of Forensic Medicine[2], Netherlands Forensic Institute, the Hague, the Netherlands

Dutch Expertise Center for Child Abuse[3], Utrecht, the Netherlands

Department of Paediatrics[4], Sophia Children's Hospital – ErasmusMC, Rotterdam, the Netherlands

Department of Paediatrics[5], Wilhelmina Children's Hospital – UMC Utrecht, Utrecht, the Netherlands

[#] Address for correspondence:
Prof. Rick R. van Rijn, MD. PhD.
Department of Radiology
Emma Children's Hospital – Amsterdam UMC, University of Amsterdam
Meibergdreef 9
1105 AZ Amsterdam Zuid-Oost
the Netherlands
Tel: 31-20-5669111
E-mail: r.r.vanrijn@amsterdamumc.nl

For peer review only - http://bmjopen.bmj.com/site/about/guidelines.xhtml

Collaborators of the Dutch Expertise Centre for Child Abuse Study Group are:

- Department of Forensic Medicine, Netherlands Forensic Institute, the Hague, the Netherlands. Collaborators: R.A.C. Bilo, MD., H.G.T. Nijs, MD. PhD., H.C. Terlingen, MD., S. de Vries, MD., N.L. van Woerden, MD. PhD.

- Department of Pediatrics, Emma Children's Hospital - Amsterdam UMC, University of Amsterdam, the Netherlands. Collaborators: L. van der Berg, MD., A.C.M. van Bellegem, MD., M. Bouman, MD., M.H. Loos, MD., A.S. Smeijers, MD., A.H. Teeuw, MD. PhD.

- Department of Pediatrics, Sophia Children's Hospital – ErasmusMC, Rotterdam, the Netherlands. Collaborators: P.J. Puiman, MD. PhD.

- Department of Pediatrics, Wilhelmina Children's Hospital – UMC Utrecht, Utrecht, the Netherlands. Collaborators: F.M.C. van Berkestijn, MD, F. Kamberg, S.L. Nijhof, MD. PhD, J.M. Ruskamp, MD. PhD., I.M.B. Russel-Kampschoer, MD., M. Schouten, MD. PhD., K. Sijstermans, MD. PhD., S.A.A. Wolt-Plompen, MD.

Keywords
Child protection
Child Abuse
Forensic Medicine

Wordcount
3120

Contributors' Statement

R.R. van Rijn collected data, carried out the initial analyses, and drafted the initial and revised manuscript, and reviewed and revised the manuscript.
M.J. Affourtit designed the data collection instruments and critically reviewed the manuscript.
W.A. Karst designed the data collection instruments and critically reviewed the manuscript.
M. Kamphuis carried out the secondary analyses, and drafted the revised manuscript, and critically reviewed and revised the manuscript.
L.C. de Bock collected data, carried out the secondary analyses and critically reviewed the manuscript
E. van de Putte designed the data collection instruments, and coordinated and supervised data collection, and critically reviewed the manuscript.

All authors approved the final manuscript as submitted and agree to be accountable for all aspects of the work.

All collaborators have read the final manuscript and agree to their name being added to the list of collaborators.

For peer review only

For peer review only - http://bmjopen.bmj.com/site/about/guidelines.xhtml

Funding Source: The Dutch Expertise Centre for Child Abuse is supported by grants from Stichting Kinderpostzegels, Nationale Postcode Loterij, Janivo Stichting, SOD & Fie van der Hoop fonds and governmental support from the Ministry of Health, Welfare and Sport.

Financial Disclosure:
All authors are (part-time or full) employees the Dutch Expertise Centre for Child Abuse.

Conflict of Interest:
The authors have no conflicts of interest to disclose.

Abbreviations:
DECCA = Dutch Expertise Centre for Child Abuse

For peer review only

For peer review only - http://bmjopen.bmj.com/site/about/guidelines.xhtml

Abstract

Objective

Combined paediatric and forensic medical expertise is not available in Dutch healthcare facilities. The Dutch Expertise Centre for Child Abuse (DECCA) was founded in 2014 through the conviction that this combination is essential in assessing potential physical child abuse. DECCA is a collaboration between three paediatric hospitals and the Netherlands Forensic Institute. DECCA works with Bayes' theorem and adds likelihood ratios in the conclusions. We present the process of implementation of DECCA and data of the first four years.

Participants

Between December 14th 2014 and December 31st 2018 a total of 761 advisory requests were referred. All advices given were anonymously recorded in a database. Also, an advisee evaluation (2015) was used to gain insight in the first experiences with DECCA.

Results

761 cases were included, 381 (51.3%) were boys and 361 (48.7%) girls. Median age was 1.5 years (range 1 day – 20 year).

Paediatricians (53.1%) and child safeguarding doctors (21.9%) most frequently contacted DECCA.

Two most common reasons for referral were presence of injury/skin lesions (n=592) and clinical history inconsistent with findings (n=145). Most common injuries were hematoma (264) and fractures other than skull fractures (166).

The outcome of DECCA evaluation was almost certainly no or improbable child abuse in 35.7%; child abuse likely or almost certain in 24.3%; and unclear (more investigation needed) in 12%.

For peer review only - http://bmjopen.bmj.com/site/about/guidelines.xhtml

The 61 advisee evaluations (response 50%) showed that 93% experienced a lot of added value and 100% was (very) satisfied with the given advice.

Conclusion

Data show growing interest in the expertise of DECCA through the years. DECCA seems to be a valuable addition to the Dutch system of child protection, since advisee appreciate the service and outcome of DECCA evaluations. In almost half of the cases DECCA concluded that child abuse could not be substantiated.

Strengths and limitations of this study

- With the growing number of advisory requests and satisfied advisees, we were able to show that organising and providing around-the-clock, expert based independent high quality combined paediatric and forensic medical expertise on a national level is feasible.
- We have shown that through interdisciplinary collaboration in an early phase of care, health care professionals can be helped in deciding whether findings are more likely the result of a disease, accidental trauma or child abuse with the use of Bayes' theorem. In those cases where additional information or expert opinion is needed, DECCA refer to specific consultants.
- As the Dutch Expertise Centre for Child Abuse works with anonymous advice no follow-up of the individual cases is possible. Therefore a health economics analysis, however important, will be difficult.
- An update of the advisee evaluations is needed and will be set up in 2019.

For peer review only

Introduction

Child abuse is a world-wide ubiquitous problem leading to both short term and long term negative outcomes in health and social well-being of the victims [1 2]. It has a detrimental impact on the development of children, with an impact on the individual throughout life. The seminal study by Fellitti et al. has shown that there is an increase of disease and early death in adulthood in child abuse survivors [1]. Besides the direct impact on the victim it has been shown to have a significant cost to society [3 4].

Both from an individual as well as a societal point of view it is important to prevent and detect child abuse as early as possible and intervene when child abuse is suspected in order to prevent further abuse and to improve the outcome of the individual victim and his/her social network.

In the Netherlands, in contrast to many other western countries, physicians are only mandated to reporter child abuse in specific cases according to the guideline Domestic violence and child abuse as published by the Royal Dutch Medical Association (figure 1) [5]. This guideline dictates five steps, of which the first step is to collect information about the patient in question and assess the potential for the presence of child abuse. If the attending physician thinks that child abuse might be present he/she is required to ask advice from or report to so-called child safeguarding doctors (step 2). Child safeguarding doctors work regionally within a national system in which all cases of possible child abuse are reported and investigated and they can, in collaboration with a wide variety of health care support teams, instigate interventions.

However, in order to substantiate potential abuse as well as reject this diagnosis in the first step of the diagnostic pathway, specifically concerning injuries on physical examination and questions concerning physical signs and symptoms, in-depth knowledge of both paediatric as

For peer review only - http://bmjopen.bmj.com/site/about/guidelines.xhtml

well as forensic medicine is required. We will refer to this combination of expertise as combined paediatric forensic medical expertise. In addition, other relevant sub-specialties and affiliated specialties such as radiology, will add to this knowledge. This adheres to the recommendation of the WHO Regional Office for Europe report on the prevention of child maltreatment, to use a multidisciplinary approach to cases that use reliable and valid investigative methods (2013) [6]. In the Netherlands, as in most countries, this broad combined range of specialties is not available in hospitals or health care facilities. Given this fact and the necessity of 24/7 support for health care professionals confronted with a potential case of child abuse, we founded the 'Landelijk Expertise Centrum Kindermishandeling' (Dutch Expertise Centre for Child Abuse (DECCA)) in 2014 [7]. DECCA can be consulted in step 1 and step 2 of the national guideline Domestic violence and child abuse [5]. Child death reviews are not part of the objectives of DECCA. In this publication we present the organisation and methods of the DECCA and our initial experiences in the first four years since its start.

Organisation of the DECCA

Advices and consultation are performed by DECCA-paediatricians and DECCA-forensic physicians. The DECCA is a tax exempted foundation initiated by three university paediatric hospitals (Emma Children's Hospital – Amsterdam UMC – University of Amsterdam, Sophia Children's Hospital – ErasmusMC Rotterdam, and Wilhelmina Children's Hospital – UMC Utrecht) and the department of forensic medicine of the Netherlands Forensic Institute, The Hague. The DECCA as such is a virtual organisation, i.e. it holds no buildings nor offices. DECCA holds a board (consisting of a physician of each centre and a treasurer), a central coordinator, a secretary and as of 2018 a medical director. In each centre one of the

For peer review only - http://bmjopen.bmj.com/site/about/guidelines.xhtml

involved physicians is in the lead and these four team leaders meet on a semi-annual basis with the board of DECCA to discuss points for improvement and future developments. In addition, an advisory board and advisors meet up with the board two to three times a year.

Method of DECCA

The DECCA is available for health care professionals seven days a week, day and night, by a national telephone number which is manned by a paediatrician with expertise and experience in child abuse diagnostics (an additional two and half years of education). At the same time a forensic physician, with expertise and experience in child abuse evaluation is on call. Specific quality criteria for the physicians working at DECCA are described and are maintained by the board and medical director of DECCA.

Advisees of the DECCA are mainly medical doctors who ask for a one-off advice (by telephone or email) or refer for consultation. Besides medical information, photographs and radiography can be provided for review. Focus is mainly on physical injury and questions concerning physical signs and symptoms. In all cases the advisee will get a combined advice from both a paediatrician and a forensic physician ensuring optimal expertise. At first a conclusion is formulated conform Bayes' theorem, that describes the probability for a hypothesis including likelihood ratios (e.g. the probability of child abuse versus the probability of a disease or accident concerning a certain injury). This conclusion is substantiated with data from scientific publications. The final advice concerning the judgement of the injury is independent of social history. The search for evidence and the discussion between the paediatrician and the forensic physician continues until they agree. In case of different opinions or lack of evidence, additional advice is obtained from other experts both nationally as well as internationally. In all cases in which DECCA is asked for advice, the advisee will receive a letter stating the anonymous information provided by the

For peer review only - http://bmjopen.bmj.com/site/about/guidelines.xhtml

advisee, the conclusions reached by DECCA and if needed the advice for further assessment or follow-up. It is specifically stated that this letter should be included in the patient's medical record. All cases are recorded in a secure on-line data base (Castor EDC®, CIWIT B.V., Amsterdam, the Netherlands) to which all DECCA professionals have password protected access to, including two-factor authentication. The database conforms to the guidelines of good clinical practice and the General Data Protection Regulation [8]. Advice will be given anonymously and personal information of the child or the family is not added to our data base. Name and email address of the advisee is included in the data base, in order to be able to send the letter of advice to the advisee.

DECCA telephone conference in which all centres are engaged and at least two senior paediatricians and a forensic physician must be present is held weekly. During this telephone conference all cases of the past week are discussed anonymously. In this discussion the paediatrician who initially handled the case presents it to the participating DECCA physicians and the advice given is discussed, including the written report. Based on the group discussion the advice can be altered. In case of an updated or altered advice the referring health care professional is contacted to discuss this update. The DECCA is not involved in further treatment or follow-up of the cases.

In those cases where additional specific expertise for physical examination or additional investigations are required, DECCA can advise referral of the patient to a paediatric centre with expertise on child abuse (including one of the three centres collaborating in the DECCA) for out-patient follow-up and evaluation. For example, this could be the case in investigation of signs of sexual abuse including securing possible DNA evidence. On average 35 cases per year are referred to one of the three academic paediatric hospitals of DECCA for face-to-face

For peer review only - http://bmjopen.bmj.com/site/about/guidelines.xhtml

consultation. These cases are part of academic patient care, are not the main focus of DECCA, and are therefore not presented in this overview.

Data collection and statistical analyses

Between December 14th 2014 and December 31st 2018 a total of 761 advisory requests were recorded by the DECCA paediatricians in the DECCA database. Additionally, an evaluation of the experiences of advisees was performed. During six months all advisees received an online survey consisting of fifteen questions about their advisory request, concerning how they experienced the added value of DECCA, if they were satisfied with the advice as such and how DECCA could improve their service. In total 122 advisees were asked to participate anonymously in an on-line questionnaire sent directly after they had received advice from May 2015 to January 2016.

Statistical analysis was performed using IBM SPSS Statistics for Windows, Version 25. (Armonk, NY: IBM Corp.). Analyses concerned the processing of frequencies or cross tables.

Patient and Public Involvement

As DECCA doesn't record patient identifiers, patients and/or their parents were not included in this descriptive study of the first four years of DECCA.

Results

Advisory requests

All cases of advisory requests recorded in the data base were included in the analyses, this concerns a total of 761. Figure 2 shows the growing number of requests during the years. There were 381 (51.3%) boys and 361 (48.7%) girls; in 19 cases sex was not disclosed. Age

For peer review only - http://bmjopen.bmj.com/site/about/guidelines.xhtml

was not recorded in 9 cases, the median age of the referred population was 1.5 years (range 1 day – 20 years). The 20 year-old, although too old according to the definition of child abuse, was a sibling of a younger child, and was therefore referred to DECCA and included in this study. The median age in boys was 1.4 years, the mean age was 2.7 years (SD 3.4 years) and in girls this was respectively 2 and 3.8 years (SD 4.5 years). The geographic distribution of the referrers was skewed with a majority of cases being referred from the western provinces of The Netherlands, corresponding to a more densely populated area. (figure 3). The cases were referred throughout the week, though mainly during weekdays. Cases were referred throughout the day and night, however mostly during office hours.

There was a wide variety of health care professionals who consulted the DECCA for advice on cases in which they had findings concerning (suspected) child abuse (table 1). Paediatricians were the most prevalent advisees (53.1%), with child safeguarding doctors as second. This is shifting over the years towards more requests from child safeguarding doctors.

In some cases expertise from other medical specialists was needed, DECCA therefore involved several clinical subspecialties in the advice process besides paediatrics and forensics, primarily paediatric radiology (table 2).

The primary question of the advisee mainly concerned ‘Does the observed injury fit the described trauma mechanism?’ and ‘Could the nature of the injury be ascertained?’ As is common in (suspected) child abuse there were often more than one findings or red flags leading to a request for DECCA advice (table 3). Most common injuries in advisory requests were hematoma (264), fractures (166), skull fractures (69) and brain damage (64) (table 4).

In addition to answering the specific question of the advisee and when possible, the conclusion of the DECCA advice specifically states a level of certainty with respect to child

For peer review only

For peer review only - http://bmjopen.bmj.com/site/about/guidelines.xhtml

abuse. Table 5 shows the outcome regarding the possibility of child abuse as concluded by the DECCA. This was almost certainly no or improbable child abuse in 35.7%; child abuse possible in 28%; and child abuse likely or almost certain in 24.3%. In 12% of cases additional investigations were advised.

Advisee evaluation:

In total 61 out of 122 (50%) advisee responded to the online evaluation questionnaire. Of these respondents 39 (64%) stated that this was the first time they contacted DECCA and 22 (36%) stated that they previously had been in contact with DECCA. In the majority of cases the respondents found that the DECCA advice had either much additional value (N=51 (84%)) or very much additional value (N=8 (13%)). Only two respondents (3%) found that the DECCA advise was of little additional value. The DECCA advice changed the perspective on the case in 33 (54%) of cases. In all cases the respondents were (very) satisfied about the DECCA advise and marked it on average as 8.4 on a scale from 0 to 10 (range from 7 to 10, with 16 respondents scoring 9 and 5 scoring 10).

Discussion

In the past four years DECCA has shown that organising and providing expert-based and independent combined paediatric and forensic medical expertise on a national level is feasible. Interest for contacting DECCA is growing nationally. From both a care providers' viewpoint as the child's viewpoint DECCA's involvement had an impact on the outcome on the next steps in the reporting process of (suspected) child abuse, because it added to, changed or confirmed the former conclusion of the advisee.

For peer review only - http://bmjopen.bmj.com/site/about/guidelines.xhtml

Teamwork in child abuse diagnostics in itself is not a novel development. In most countries hospitals are required to have some form of a hospital-based multidisciplinary team, and these have shown their value over time [9-11]. Besides these hospital based teams many countries, as well as The Netherlands, have initiated regional or citywide rape and sexual abuse counselling centres, where in many cases specially trained sexual assault nurse examiners (SANE) and/or child abuse paediatricians work [12-14]. These centres play an important role in the diagnosis of sexual child abuse and the care for the victims thereof. The unique aspect of DECCA is the combination of both paediatric and forensic knowledge, outside the field of justice, continuously available on a nationwide scale. Each speciality brings their own expertise. The paediatrician's main focus is on the medical differential diagnosis, whereas the forensic physician adds an objective assessment of the injury and the potential causative mechanism. In addition, the use of Bayes' theorem adds to the perspective of the advisee. This combination of expertise and method yields an integral analysis of the patient and his/her injury from two different viewpoints leading to an objective and evidence based analysis of suspected child abuse, with reference to relevant literature. To our knowledge this makes DECCA unique in the world.

It has been shown, that expert consultation has added value to patient care in cases of suspected child abuse [10 15 16]. However, Linberg et al. showed that even between child abuse paediatricians there was a significant variability in assessing the likelihood of child abuse [17]. They concluded that their data supported the use of peer-review or multidisciplinary teams. The weekly telephone conference, in which all DECCA cases are discussed in-depth, clearly addresses this issue. Not only does this provide a unique opportunity to share knowledge

For peer review only - http://bmjopen.bmj.com/site/about/guidelines.xhtml

and learn from one another, but more important, we strongly believe that our approach increases the quality and consistency of the advice given to the referrer.

A limitation of this study is that, despite its undeniable value, we could not do a health economics analysis. Long term follow-up of (a subset of) cases will be extremely difficult especially since we register our cases anonymously. With the introduction of the General Data Protection Regulation (GDPR), follow-up would only be possible with consent from both caregivers/parents/legal guardians. This would almost certainly introduce an inclusion bias. No inter-rater reliability assessment is done. However, we restructured our database and as of 2019 we register inconsistencies between the first advice and the joint assessment during the weekly peer-review. This will eventually give us insight in potential shortcomings in our DECCA advice. In addition, the advisee evaluation has been restructured where advisees are requested to fill out a short questionnaire after each consultation. These two procedural changes will form the basis of a new quality assessment.

A major difficulty of diagnosing child abuse lays in one of the principal precepts of bioethics we teach all our medical students: 'primum non nocere'. In cases of potential child abuse the clinician must weigh the risk of underreporting versus overreporting, as both can have serious consequences for the child and its caregivers. As a result it is well known that, even in countries with mandated reporting, there is a significant level of underreporting of child abuse and neglect. One of the explanations for underreporting is reported to be a lack of knowledge leading to uncertainty of the diagnosis of child abuse [18-20]. It could be argued that if expertise centres like DECCA are more widely available this could lead to an increase in

For peer review only - http://bmjopen.bmj.com/site/about/guidelines.xhtml

For peer review only

diagnosing and reporting child abuse, but also in rejecting the diagnosis child abuse in an earlier phase of the diagnostic process.

The data we present have a significant drawback. As DECCA acts in the first or second step of the guideline as published by the Royal Dutch Medical Association and mainly focusses on findings during the physical examination, there is a distinct referral bias. As a result, cases where physical or sexual child abuse are suspected are overrepresented in our study population in comparison to other child abuse studies. The role of a national paediatric forensic medical expertise centre will in most cases be limited to these two types of child abuse, where physical injury and physical signs are most evident. For cases in which neglect or deprivation play an important role, without signs of physical injury, a face-to-face clinical consultation with a paediatrician and/ or child psychologist with expertise in this field remains the best solution. The strong involvement of three paediatric university hospitals guarantees that in forthcoming cases this service can also be offered to referring health care professionals.

The main challenge the DECCA has faced was how to get its work financed. As the DECCA works with anonymous cases it therefore cannot bill the patient's insurance company. A grant from two Dutch charity funds, Stichting Kinderpostzegels and Nationale Postcode Loterij, made it possible to start DECCA in 2014 and paid for the running costs for the first year. Currently the Dutch Ministry of Health, Welfare and Sport fully finances our work on a yearly basis. There are no studies into the economic added value of services like the DECCA. However, a study into the value of telephone consultations of paediatric subspecialists to primary care physicians was shown to be cost-effective as a result of reduced use of costly

For peer review only - http://bmjopen.bmj.com/site/about/guidelines.xhtml

services and reported improvements in quality of care [21]. We firmly believe that the DECCA has a similar impact on patient care and being the sole provider of this care in the Netherlands, the DECCA should be financed on a long-term project basis. This financial issue will play a role in other countries as well and could limit the implementation of similar models elsewhere.

Conclusion

The Dutch Expertise Centre for Child Abuse seems to be a valuable addition to the Dutch system of child protection based on the rising number of advisory requests and satisfied advisees. By virtue of its virtual centralised design this model could, in an adapted form, be established in other countries as well. We are convinced that the combination of experts in the field of paediatrics and forensic medicine including working with Bayes' theorem, and working together with affiliated (sub-)specialties, enables to provide optimal, evidence-based paediatric forensic medical expertise.

For peer review only

For peer review only - http://bmjopen.bmj.com/site/about/guidelines.xhtml

1 **Table 1. Overview of advisees to DECCA (n=761).**

Specialty	N	% of all 761 cases
Paediatrician	404	53.1
Child safeguarding doctors	167	21.9
General practitioner	20	2.6
Youth health care doctor	12	1.6
Emergency Room doctor	9	1.2
Youth care worker	7	0.9
Others (like surgeons, general practitioners or paediatricians in training, psychiatrist, dentist, forensic nurses at ER, nurse practitioners)	142	18.7

2

For peer review only

For peer review only - http://bmjopen.bmj.com/site/about/guidelines.xhtml

Table 2.

Subspecialties involved in DECCA (n=761).

Subspecialty	N[1]	% of all 761 cases
Paediatric radiology	296	38.9
Paediatric dermatology	31	4.1
Paediatric ophthalmology	22	2.9
Paediatric neurology	12	1.6
Paediatric haematology	7	0.9
Social work	7	0.9
Paediatric surgery	3	0.4
Others (like paediatric urology, ear-nose-throat doctor, paediatric immunology)	15	2.0

[1] The total does not sum up to 761 as not for every case consultant of a subspecialty is needed and several subspecialties can be consulted in one single case.

For peer review only

Table 3. Findings or red flags leading to a request for DECCA advice

Finding or red flag	N[1]
Injury/ skin lesion	592
Clinical history not in keeping with findings	145
Presence of risk factors in family	70
Physical symptoms	71
Inconsistent clinical history	47
Lesion/trauma not in keeping with child's age	40
Delay in presentation	39
Previous trauma	33
Child admits being a victim of abuse	26
Behavioural symptoms child	19
Caregiver admits to child abuse	17
Improper hygiene child	9
Inadequate interaction child – caregiver	8
Other child in family discloses about child abuse	8
Findings at additional investigations (urine- or bloodtest)	2

[1] The total does not sum up to 761 as several findings or red flags can be present in one single case and several findings can be mentioned in one single case.

For peer review only

Table 4. Four most common injuries in advisory requests

Most common mentioned type of Injury	N[1]
Haematoma	264
Fractures other than skull fracture	166
Skull fracture	69
Brain damage	64

[1] The total does not sum up to 761 as several findings or red flags can be present in one single case and not all injuries are being listed here.

For peer review only

For peer review only - http://bmjopen.bmj.com/site/about/guidelines.xhtml

Table 5. Outcome of DECCA evaluation (N=753, 8 missing)

**Outcome of DECCA evaluation**	**Number**	**% of 753 cases**
Almost certainly no child abuse	84	11.1
Child abuse unlikely	185	24.6
Child abuse possible	211	28.0
Child abuse likely	114	15.1
Almost certainly child abuse	69	9.2
Unclear, more investigation needed	90	12.0

For peer review only

For peer review only - http://bmjopen.bmj.com/site/about/guidelines.xhtml

Legend to figures

Figure 1. Stepwise approach of managing child abuse as guideline as published by the Royal Dutch Medical Association [5].

Figure 2. Number of DECCA advisory requests from 2015 to 2018.

Figure 3. Geographic distribution of the advisee to the Dutch Expertise Centre for Child Abuse (N= 740, 21 missing).

For peer review only

For peer review only - http://bmjopen.bmj.com/site/about/guidelines.xhtml

References

1. Felitti VJ, Anda RF, Nordenberg D, et al. Relationship of childhood abuse and household dysfunction to many of the leading causes of death in adults. The Adverse Childhood Experiences (ACE) Study. *Am J PrevMed* 1998;14(4):245-58.

2. Gilbert R, Widom CS, Browne K, et al. Burden and consequences of child maltreatment in high-income countries. *Lancet* 2009;373(9657):68-81. doi: 10.1016/s0140-6736(08)61706-7 [published Online First: 2008/12/06]

3. Irazuzta JE, McJunkin JE, Danadian K, et al. Outcome and cost of child abuse. *Child Abuse Negl* 1997;21(8):751-57.

4. Gelles RJ, Perlman S. Estimated Annual Cost of Child Abuse and Neglect. Chicago IL: Prevent Child Abuse America,, 2012.

5. Royal Dutch Medical Association. KNMG-meldcode kindermishandeling en huiselijk geweld 2018 [Available from: https://www.knmg.nl/advies-richtlijnen/knmg-publicaties/meldcode-kindermishandeling-en-huiselijk-geweld-1.htm.

6. WHO Regional Office for Europe. European report on preventing child maltreatment. In: Sethi D, Bellis M, Hughes K, et al., eds., 2013.

7. Kindermishandeling LEC. [Available from: www.leck.nu accessed 5-10-2017.

8. European Parliament. 2018 reform of EU data protection rules 2018 [Available from: https://ec.europa.eu/commission/priorities/justice-and-fundamental-rights/data-protection/2018-reform-eu-data-protection-rules_en accessed 10-4-2019.

9. Brink FW, Thackeray JD, Bridge JA, et al. Child advocacy center multidisciplinary team decision and its association to child protective services outcomes. *Child Abuse Negl*

For peer review only - http://bmjopen.bmj.com/site/about/guidelines.xhtml

2015;46:174-81. doi: 10.1016/j.chiabu.2015.04.011 [published Online First: 2015/05/11]

10. Draus JM, Jr. A Multidisciplinary Child Protection Team Improves the Care of Nonaccidental Trauma Patients. *The American surgeon* 2017;83(5):477-81. [published Online First: 2017/05/26]

11. Wallace GH, Makoroff KL, Malott HA, et al. Hospital-based multidisciplinary teams can prevent unnecessary child abuse reports and out-of-home placements. *Child Abuse Negl* 2007;31(6):623-9. doi: 10.1016/j.chiabu.2006.12.010 [published Online First: 2007/06/26]

12. Bechtel K, Ryan E, Gallagher D. Impact of sexual assault nurse examiners on the evaluation of sexual assault in a pediatric emergency department. *Pediatric emergency care* 2008;24(7):442-7. doi: 10.1097/PEC.0b013e31817de11d [published Online First: 2008/06/27]

13. Adams JA, Starling SP, Frasier LD, et al. Diagnostic accuracy in child sexual abuse medical evaluation: role of experience, training, and expert case review. *Child Abuse Negl* 2012;36(5):383-92. doi: 10.1016/j.chiabu.2012.01.004 [published Online First: 2012/05/29]

14. Hornor G, Thackeray J, Scribano P, et al. Pediatric sexual assault nurse examiner care: trace forensic evidence, ano-genital injury, and judicial outcomes. *Journal of forensic nursing* 2012;8(3):105-11. doi: 10.1111/j.1939-3938.2011.01131.x [published Online First: 2012/08/29]

15. McGuire L, Martin KD, Leventhal JM. Child abuse consultations initiated by child protective services: the role of expert opinions. *Academic pediatrics* 2011;11(6):467-73. doi: 10.1016/j.acap.2011.06.005 [published Online First: 2011/08/09]

For peer review only - http://bmjopen.bmj.com/site/about/guidelines.xhtml

16. Teeuw AH, Sieswerda-Hoogendoorn T, Aaftink D, et al. Assessments carried out by a child abuse and neglect team in an Amsterdam teaching hospital led to interventions in most of the reported cases. *Acta paediatrica (Oslo, Norway : 1992)* 2017;106(7):1118-27. doi: 10.1111/apa.13735 [published Online First: 2016/12/31]

17. Lindberg DM, Lindsell CJ, Shapiro RA. Variability in expert assessments of child physical abuse likelihood. *Pediatrics* 2008;121(4):e945-53. doi: 10.1542/peds.2007-2485 [published Online First: 2008/04/03]

18. Sege R, Flaherty E, Jones R, et al. To report or not to report: examination of the initial primary care management of suspicious childhood injuries. *Academic pediatrics* 2011;11(6):460-6. doi: 10.1016/j.acap.2011.08.005 [published Online First: 2011/10/15]

19. Talsma M, Bengtsson Bostrom K, Ostberg AL. Facing suspected child abuse--what keeps Swedish general practitioners from reporting to child protective services? *Scandinavian journal of primary health care* 2015;33(1):21-6. doi: 10.3109/02813432.2015.1001941 [published Online First: 2015/02/14]

20. Vulliamy AP, Sullivan R. Reporting child abuse: pediatricians' experiences with the child protection system. *Child Abuse Negl* 2000;24(11):1461-70. [published Online First: 2000/12/29]

21. Wegner SE, Humble CG, Feaganes J, et al. Estimated savings from paid telephone consultations between subspecialists and primary care physicians. *Pediatrics* 2008;122(6):e1136-40. doi: 10.1542/peds.2008-0432 [published Online First: 2008/12/03]

For peer review only

For peer review only - http://bmjopen.bmj.com/site/about/guidelines.xhtml

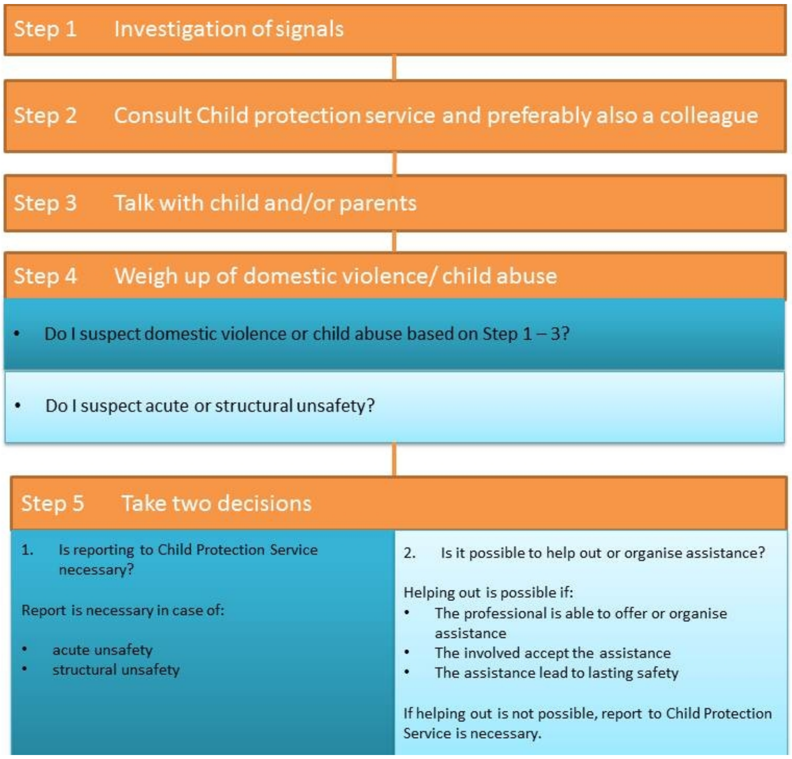

Stepwise approach of managing child abuse as guideline as published by the Royal Dutch Medical Association 5.

254x190mm (96 x 96 DPI)

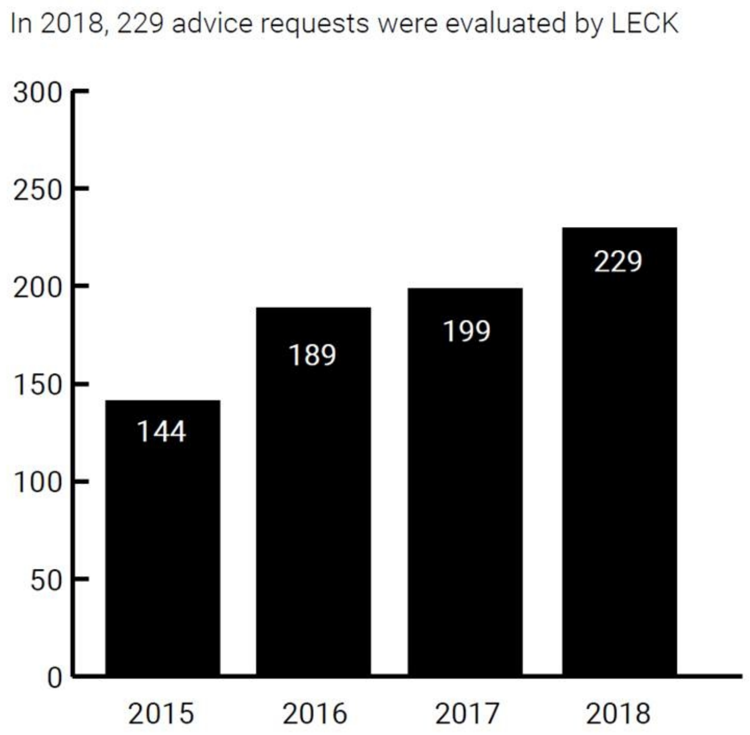

Number of DECCA advisory requests from 2015 to 2018.

254x190mm (96 x 96 DPI)

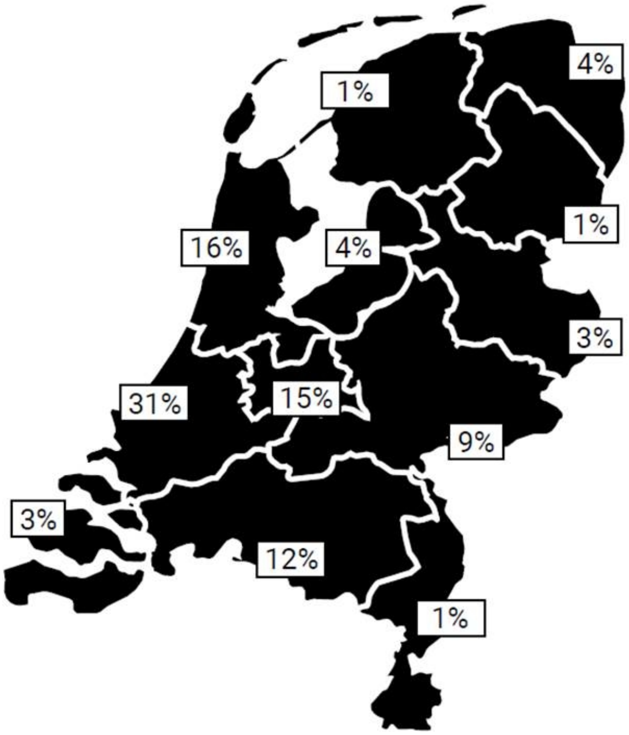

Geographic distribution of the advisee to the Dutch Expertise Centre for Child Abuse (N= 740, 21 missing).

254x190mm (96 x 96 DPI)

STROBE Statement - The start of the Dutch Expertise Centre for Child Abuse; a descriptive study of the implementation and data of the first 4 years

	Item	Recommendation	Page
**Title and abstract**	1	(*a*) Indicate the study's design with a commonly used term in the title or the abstract	Title page
		(*b*) Provide in the abstract an informative and balanced summary of what was done and what was found	2
**Introduction**			
Background/rationale	2	Explain the scientific background and rationale for the investigation being reported	4
Objectives	3	State specific objectives, including any prespecified hypotheses	5
**Methods**			
Study design	4	Present key elements of study design early in the paper	8
Setting	5	Describe the setting, locations, and relevant dates, including periods of recruitment, exposure, follow-up, and data collection	8
Participants	6	(*a*) *Cohort study*—Give the eligibility criteria, and the sources and methods of selection of participants. Describe methods of follow-up *Case-control study*—Give the eligibility criteria, and the sources and methods of case ascertainment and control selection. Give the rationale for the choice of cases and controls *Cross-sectional study*—Give the eligibility criteria, and the sources and methods of selection of participants	8
		(*b*) *Cohort study*—For matched studies, give matching criteria and number of exposed and unexposed *Case-control study*—For matched studies, give matching criteria and the number of controls per case	N/A
Variables	7	Clearly define all outcomes, exposures, predictors, potential confounders, and effect modifiers. Give diagnostic criteria, if applicable	N/A
Data sources/ measurement	8	For each variable of interest, give sources of data and details of methods of assessment (measurement). Describe comparability of assessment methods if there is more than one group	N/A
Bias	9	Describe any efforts to address potential sources of bias	N/A
Study size	10	Explain how the study size was arrived at	N/A
Quantitative variables	11	Explain how quantitative variables were handled in the analyses. If applicable, describe which groupings were chosen and why	N/A
Statistical methods	12	(*a*) Describe all statistical methods, including those used to control for confounding	8
		(*b*) Describe any methods used to examine subgroups and interactions	N/A
		(*c*) Explain how missing data were addressed	N/A
		(*d*) *Cohort study*—If applicable, explain how loss to follow-up was addressed *Case-control study*—If applicable, explain how matching of cases and controls was addressed	N/A

For peer review only

		Cross-sectional study—If applicable, describe analytical methods taking account of sampling strategy	
		(*e*) Describe any sensitivity analyses	N/A
Results			
Participants	13*	(a) Report numbers of individuals at each stage of study—eg numbers potentially eligible, examined for eligibility, confirmed eligible, included in the study, completing follow-up, and analysed	N/A
		(b) Give reasons for non-participation at each stage	N/A
		(c) Consider use of a flow diagram	N/A
Descriptive data	14*	(a) Give characteristics of study participants (eg demographic, clinical, social) and information on exposures and potential confounders	8
		(b) Indicate number of participants with missing data for each variable of interest	N/A
		(c) *Cohort study*—Summarise follow-up time (eg, average and total amount)	N/A
Outcome data	15*	*Cohort study*—Report numbers of outcome events or summary measures over time	N/A
		Case-control study—Report numbers in each exposure category, or summary measures of exposure	N/A
		Cross-sectional study—Report numbers of outcome events or summary measures	N/A
Main results	16	(*a*) Give unadjusted estimates and, if applicable, confounder-adjusted estimates and their precision (eg, 95% confidence interval). Make clear which confounders were adjusted for and why they were included	N/A
		(*b*) Report category boundaries when continuous variables were categorized	N/A
		(*c*) If relevant, consider translating estimates of relative risk into absolute risk for a meaningful time period	N/A
Other analyses	17	Report other analyses done—eg analyses of subgroups and interactions, and sensitivity analyses	N/A
**Discussion**			
Key results	18	Summarise key results with reference to study objectives	10
Limitations	19	Discuss limitations of the study, taking into account sources of potential bias or imprecision. Discuss both direction and magnitude of any potential bias	12
Interpretation	20	Give a cautious overall interpretation of results considering objectives, limitations, multiplicity of analyses, results from similar studies, and other relevant evidence	11
Generalisability	21	Discuss the generalisability (external validity) of the study results	11
**Other information**			
Funding	22	Give the source of funding and the role of the funders for the present study and, if applicable, for the original study on which the present article is based	13

For peer review only

For peer review only - http://bmjopen.bmj.com/site/about/guidelines.xhtml

Implementation of the Dutch Expertise Centre for Child Abuse: descriptive data from the first four years

Journal:	*BMJ Open*
Manuscript ID	bmjopen-2019-031008.R1
Article Type:	Research
Date Submitted by the Author:	18-Jun-2019

Complete List of Authors:	van Rijn, Rick; Emma Children's hospital, Academic Medical Center, radiology; Nederlands Forensisch Instituut, Forensic Medicine<br>Affourtit, Marjo; Erasmus MC Sophia Children's Hospital, Pediatrics<br>Karst, Wouter; Nederlands Forensisch Instituut, Forensic Medicine<br>Kamphuis, Mascha; Dutch Expertise Center for Child Abuse<br>de Bock, Leonie; Dutch Expertise Center for Child Abuse<br>van de Putte, Elise; University Medical Center Utrecht, General Paediatrics

**Primary Subject Heading**:	Paediatrics
Secondary Subject Heading:	Paediatrics
Keywords:	Child protection < PAEDIATRICS, Child Abuse, FORENSIC MEDICINE

SCHOLARONE™ Manuscripts

Implementation of the Dutch Expertise Centre for Child Abuse: descriptive data from the first four years

R.R. van Rijn[1,2#,3], M.J. Affourtit[3,4], W.A. Karst[2,3], M. Kamphuis[3], L.C. de Bock[3], E. van de Putte[3,5], on behalf of the Dutch Expertise Centre for Child Abuse Study Group

Department of Radiology[1], Emma Children's Hospital - Amsterdam UMC, University of Amsterdam, Amsterdam, the Netherlands

Department of Forensic Medicine[2], Netherlands Forensic Institute, the Hague, the Netherlands

Dutch Expertise Center for Child Abuse[3], Utrecht, the Netherlands

Department of Paediatrics[4], Sophia Children's Hospital – ErasmusMC, Rotterdam, the Netherlands

Department of Paediatrics[5], Wilhelmina Children's Hospital – UMC Utrecht, Utrecht, the Netherlands

[#] Address for correspondence:
Prof. Rick R. van Rijn, MD. PhD.
Department of Radiology and Nuclear Medicine
Emma Children's Hospital – Amsterdam UMC, University of Amsterdam
Meibergdreef 9
1105 AZ Amsterdam Zuid-Oost
the Netherlands
Tel: 31-20-5669111
E-mail: r.r.vanrijn@amc.uva.nl

For peer review only

For peer review only - http://bmjopen.bmj.com/site/about/guidelines.xhtml

Collaborators of the Dutch Expertise Centre for Child Abuse Study Group are:

- Department of Forensic Medicine, Netherlands Forensic Institute, the Hague, the Netherlands. Collaborators:
    - R.A.C. Bilo, MD.
    - H.G.T. Nijs, MD. PhD.
    - H.C. Terlingen, MD.
    - S. de Vries, MD.
    - N.L. van Woerden, MD. PhD.

- Department of Paediatrics, Emma Children's Hospital - Amsterdam UMC, University of Amsterdam, the Netherlands, Collaborators:
    - L. van der Berg, MD.
    - A.C.M. van Bellegem, MD.
    - M. Bouman, MD.
    - M.H. Loos, MD.
    - A.S. Smeijers, MD.
    - A.H. Teeuw, MD. PhD.

- Department of Paediatrics, Sophia Children's Hospital – ErasmusMC, Rotterdam, the Netherlands. Collaborators:
    - P.J. Puiman, MD. PhD.

- Department of Paediatrics, Wilhelmina Children's Hospital – UMC Utrecht, Utrecht, the Netherlands. Collaborators:
    - F.M.C. van Berkestijn, MD.
    - F. Kamberg
    - S.L. Nijhof, MD. PhD.
    - J.M. Ruskamp, MD. PhD.
    - I.M.B. Russel-Kampschoer, MD.
    - M. Schouten, MD. PhD.
    - K. Sijstermans, MD. PhD.
    - S.A.A. Wolt-Plompen, MD.

Keywords
Child protection
Child Abuse
Forensic Medicine

Wordcount
3843

Abbreviations:
DECCA = Dutch Expertise Centre for Child Abuse

For peer review only - http://bmjopen.bmj.com/site/about/guidelines.xhtml

Abstract

Objective

Combined paediatric and forensic medical expertise to interpret physical findings is not available in Dutch healthcare facilities. The Dutch Expertise Centre for Child Abuse (DECCA) was founded in the conviction that this combination is essential in assessing potential physical child abuse. DECCA is a collaboration between three paediatric hospitals and the Netherlands Forensic Institute. DECCA works with Bayes' theorem and uses likelihood ratios in their conclusions.

Design

We present the implementation process of DECCA and cross sectional data of the first four years.

Participants

Between December 14th 2014 and December 31st 2018 a total of 761 advisory requests were referred, all of which were included in this study. An advisee evaluation over the year 2015 was performed using a self-constructed survey to gain insight in the first experiences with DECCA.

Results

761 cases were included, 381 (50.1%) boys and 361 (47.4%) girls (19 cases (2.5%) sex undisclosed). Median age was 1.5 years (range 1 day – 20 year).

Paediatricians (53.1%) and child safeguarding doctors (21.9%) most frequently contacted DECCA.

Two most common reasons for referral were presence of injury/skin lesions (n=592) and clinical history inconsistent with findings (n=145). The most common injuries were bruises (264) and fractures other than skull fractures (166).

For peer review only - http://bmjopen.bmj.com/site/about/guidelines.xhtml

Outcome of DECCA evaluation was almost certainly no or improbable child abuse in 35.7%; child abuse likely or almost certain in 24.3%; and unclear in 12%.

The advisee evaluations (response rate 50%) showed that 93% experienced added value and that 100% were (very) satisfied with the advice.

Conclusion

Data show growing interest in the expertise of DECCA through the years. DECCA seems to be a valuable addition to Dutch child protection, since advisee value the service and outcome of DECCA evaluations. In almost half of the cases DECCA concluded that child abuse could not be substantiated.

For peer review only - http://bmjopen.bmj.com/site/about/guidelines.xhtml

Strengths and limitations of this study

- After four years of advisory requests, we have collected a large number of cases to show solid results. With the growing number of advisory requests and satisfied advisees, we were able to show that organising and providing round-the-clock, expert based independent high quality combined paediatric and forensic medical expertise on a national level is feasible.
- During the initial discussion between the paediatrician, who has spoken with the advisee, and the forensic physician, the latter is blinded with respect to the social history and other risk factors. This way we aim to avoid cognitive bias as much as possible.
- As the Dutch Expertise Centre for Child Abuse works with anonymous advice follow-up of the individual cases is not possible. Therefore a health economics analysis, although important, is impossible.
- The results of the advisee evaluations could be biased, because possibly only satisfied advisees responded to the survey.
- An update of the advisee evaluations is needed and this has been implemented as a routine part of our protocol since 1-1-2019.

For peer review only

For peer review only - http://bmjopen.bmj.com/site/about/guidelines.xhtml

Introduction

Child abuse is a world-wide ubiquitous problem leading to both short term and long term negative outcomes in health and social well-being of the victims [1 2]. It has a detrimental impact on the development of children, with an impact on the individual throughout life. The seminal study by Fellitti et al. has shown that there is an increase of disease and early death in adulthood in child abuse survivors [1]. Besides the direct impact on the victim, it has been shown to have a significant cost to society [3 4].

Both from an individual as well as a societal point of view it is important to prevent and detect child abuse as early as possible and intervene when child abuse is suspected in order to prevent further abuse and to improve the outcome of the individual victim and his/her social network.

In the Netherlands, in contrast to many other western countries, physicians are only mandated to report child abuse in specific cases according to the guideline Domestic violence and child abuse as published by the Royal Dutch Medical Association (figure 1) [5]. This guideline dictates five steps, of which the first step is to collect information about the patient in question and assess the potential for the presence of child abuse. If the attending physician thinks that child abuse might be present he/she is required to ask advice from or report to so-called child safeguarding doctors (step 2). Child safeguarding doctors work regionally within a national system in which all cases of possible child abuse are reported and investigated and they can, in collaboration with a wide variety of health care support teams, instigate interventions.

However, in order to substantiate potential abuse as well as reject this diagnosis in the first step of the diagnostic pathway, specifically concerning injuries on physical examination and questions concerning physical signs and symptoms, in-depth knowledge of both paediatric as

For peer review only - http://bmjopen.bmj.com/site/about/guidelines.xhtml

well as forensic medicine is required. We will refer to this combination of expertise as combined paediatric forensic medical expertise. In addition, other relevant sub-specialties and affiliated specialties such as radiology, will add to this knowledge. This adheres to the recommendation of the WHO Regional Office for Europe report on the prevention of child maltreatment, to use a multidisciplinary approach of cases that use reliable and valid investigative methods (2013) [6]. In the Netherlands, as in most countries, this broad combined range of specialties is not available in hospitals or health care facilities. Given this fact and the necessity for 24/7 support for health care professionals confronted with a potential case of child abuse, we founded the 'Landelijk Expertise Centrum Kindermishandeling' (Dutch Expertise Centre for Child Abuse (DECCA)) in 2014 [7]. DECCA can be consulted in step 1, step 2, and step 5 of the national guideline Domestic violence and child abuse [5]. Child death reviews are not part of the objectives of DECCA. In this publication we present the organisation and methods of the DECCA and our initial experiences in the first four years.

Organisation of the DECCA

Advice and consultations are performed by DECCA-paediatricians and DECCA-forensic physicians. The DECCA is a tax exempted foundation initiated by three university paediatric hospitals (Emma Children's Hospital – Amsterdam UMC – University of Amsterdam, Sophia Children's Hospital – ErasmusMC Rotterdam, and Wilhelmina Children's Hospital – UMC Utrecht) and the department of forensic medicine of the Netherlands Forensic Institute, The Hague. The DECCA as such is a virtual organisation: it has no buildings. DECCA is supervised by a board (consisting of a physician from each centre and a treasurer), a central coordinator, a secretary and since 2018 a medical director. In each centre one of the

For peer review only - http://bmjopen.bmj.com/site/about/guidelines.xhtml

involved physicians is in the lead and these four team leaders meet on a semi-annual basis with the board of DECCA to discuss points for improvement and future developments. In addition, an advisory board and advisors meet up with the board two to three times a year.

Method of DECCA

The DECCA is available for health care professionals seven days a week, day and night, by a national telephone number which is manned by a paediatrician with expertise and experience in child abuse diagnostics (an additional two and half years of education). At the same time a forensic physician, with expertise and experience in child abuse evaluation is on call. Specific quality criteria for the physicians working at DECCA are described and are maintained by the board and medical director of DECCA.

Advisees of the DECCA are mainly medical doctors who ask for a one-off advice (by telephone or email) or refer for consultation. Besides medical information, photographs and radiography can be provided for review. Focus is mainly on physical injury and questions concerning physical signs and symptoms. For all cases where advice is requested DECCA records the patient's data anonymously. Because of this DECCA has a low threshold for physicians, as in the Netherlands anonymous advice can be asked without permission from parents or caregivers.

In all cases the advisee will get a combined advice from both a paediatrician and a forensic physician. During the initial discussion between the paediatrician, who has spoken with the advisee, and the forensic physician, the latter is blinded with respect to the social history and other risk factors. This way we aim to avoid cognitive bias as much as possible. At first a conclusion is formed using a likelihood ratio (, that is the likelihood that the medical findings or injuries would be expected in an abused child compared to a non-abused child). This

For peer review only - http://bmjopen.bmj.com/site/about/guidelines.xhtml

conclusion is substantiated with data from scientific publications. To determine the likelihood of abuse, all other information (apart from the injuries) is used to estimate a prior probability of abuse. Hence the injuries are not used to determine the prior probability of abuse, they are only used to determine the likelihood ratio. Using Bayesian reasoning, the prior odds of abuse can be multiplied by the likelihood ratio that was determined by the forensic physician who was blinded for risk factors of abuse. This way, cognitive bias will be avoided as much as possible.

The search for evidence and the discussion between the paediatrician and the forensic physician continues until they agree. In case of different opinions or lack of evidence, additional advice is obtained from other experts both nationally as well as internationally. In all cases in which DECCA is asked for advice, the advisee will receive a letter stating the anonymous information provided by the advisee, the conclusions reached by DECCA and if needed the advice for further assessment or follow-up. It is specifically stated that this letter should be included in the patient's medical record. All cases are recorded in a secure on-line data base (Castor EDC®, CIWIT B.V., Amsterdam, the Netherlands) to which all DECCA professionals have password protected access to, including two-factor authentication. The database conforms to the guidelines of good clinical practice and the General Data Protection Regulation [8]. Advice will be given anonymously and personal information of the child or the family is not added to our data base. Name and email address of the advisee is included in the data base, in order to be able to send the letter of advice to the advisee.

A DECCA case-review telephone conference in which all centres are engaged and at least two senior paediatricians and a forensic physician must be present is held weekly. During this telephone conference all cases of the past week are discussed anonymously. In this discussion the paediatrician who initially handled the case presents it to the participating

For peer review only - http://bmjopen.bmj.com/site/about/guidelines.xhtml

DECCA physicians and the advice given is discussed, including the written report. Based on the group discussion the advice can be altered. In case of an updated or altered advice the referring health care professional is contacted to discuss this update. The DECCA is not involved in further treatment or follow-up of the cases.

In those cases in which additional specific expertise for physical examination or additional investigations is required, DECCA can advise referral of the patient to a paediatric centre with expertise on child abuse (including one of the three centres collaborating in the DECCA) for out-patient follow-up and evaluation. For example, this could be the case in investigation of signs of sexual abuse. On average 35 cases per year are referred to one of the three academic paediatric hospitals of DECCA for face-to-face consultation. These cases are part of academic patient care, hence not the main focus of DECCA, and are therefore not presented in this overview.

Methods: Data collection and statistical analyses

Between December 14th 2014 and December 31st 2018 a total of 761 advisory requests were recorded by the DECCA paediatricians in the DECCA database.

Additionally, an evaluation of the experiences of advisees was performed. This survey was specifically developed for this study and is available (in Dutch) upon request. All advisees that consulted DECCA from December 2014 until December 2015 received an online survey consisting of fifteen questions about their advisory request. This related to how they experienced the added value of DECCA, if they were satisfied with the advice as such, and how DECCA could improve their service. This evaluation was done from May 2015 to January 2016. During this time three reminders were send. In total 122 advisees were asked to

For peer review only - http://bmjopen.bmj.com/site/about/guidelines.xhtml

participate anonymously in an on-line questionnaire sent directly after they had received advice.

Statistical analysis was performed using IBM SPSS Statistics for Windows, Version 25. (Armonk, NY: IBM Corp.). Analyses involved the processing of frequencies or cross tables.

Patient and Public Involvement

As DECCA does not record patient identifiers, patients and/or their parents could not be included in this descriptive study of the first four years of DECCA.

Ethical approval

The internal review board of the University Medical Centre Utrecht, where DECCA has a postal address, issued a waiver for the documentation of consent and approved the use of an anonymized database.

Results

Advisory requests

All cases of advisory requests recorded in the data base were included in the analyses, this concerns a total of 761. Figure 2 shows the increasing number of requests during the years. There were 381 (50.1%) boys and 361 (47.4%) girls; in 19 cases (2.5%) sex was not disclosed. Age was not recorded in 9 cases. The median age of the referred population was 1.5 years (IQR 3.67, range 1 day – 20 years). The oldest case was a 20 year-old patient who, although too old according to the definition of child abuse, was a sibling of a younger child and was therefore referred to DECCA and included in this study. The median age in boys was 1.4 years (IQR 3.06) and in girls this was 2 years (IQR 5.35). The majority of cases was referred

For peer review only

from the western provinces of The Netherlands, corresponding to a more densely populated area (figure 3). The cases were referred throughout the week, though mainly during weekdays (91.9%). Cases were referred throughout the day and night, however mostly during office hours (75.9%).

There was a wide variety of health care professionals who consulted the DECCA for advice on cases in which they had findings concerning (suspected) child abuse (table 1). Paediatricians were the most prevalent advisees (53.1%), with child safeguarding doctors coming second. This is shifting over the years towards relatively more requests from child safeguarding doctors (from 21% in 2015 to 26% in 2018) and relatively less from paediatricians (from 59% in 2015 to 52% in 2018).

In some cases expertise from other medical specialists was needed, DECCA therefore involved several clinical subspecialties in the advice process besides paediatrics and forensics, primarily paediatric radiology (table 2).

The primary question of the advisee mainly concerned 'Does the observed injury fit the described trauma mechanism?' and 'Could the nature of the injury be ascertained?'. As is common in (suspected) child abuse there were often more than one finding or so-called red flags leading to a request for DECCA advice (table 3). Most common injuries in advisory requests were bruises (264), fractures (166), skull fractures (69) and brain injury (64) (table 4).

In addition to answering the specific question of the advisee and when possible, the conclusion of DECCA advice specifically states a level of certainty with respect to child abuse. Table 5 shows the outcome regarding the possibility of child abuse as concluded by DECCA. This was almost certainly no or improbable child abuse in 35.7%; child abuse possible in 28%;

For peer review only - http://bmjopen.bmj.com/site/about/guidelines.xhtml

and child abuse likely or almost certain in 24.3%. In 12% of cases additional investigations were advised.

Advisee evaluation:

In total 61 out of 122 (50%) advisees responded to the online evaluation questionnaire. Of these respondents 39 (64%) stated that this was the first time they contacted DECCA and the rest (36%) stated that they had previously been in contact with DECCA. In the majority of cases the respondents found that the DECCA advice had either much additional value (N=51 (84%)) or very much additional value (N=8 (13%)). Only two respondents (3%) found that the DECCA advice was of little additional value. The DECCA advice changed the perspective on the case in 33 (54%) of cases. In all cases the respondents were satisfied or very satisfied about the DECCA advise and marked it on average as 8.4 on a scale from 0 to 10 (range from 7 to 10, with 16 respondents scoring 9 and 5 scoring 10).

Discussion

Interest for contacting DECCA is growing nationally, as is shown by the increasing numbers of advisory requests (from 132 in 2015 to 229 in 2018). In the past four years DECCA has shown that organising and providing expert-based and independent combined paediatric and forensic medical expertise on a national level is feasible. From both a care providers' as well as the child's viewpoint DECCA's involvement had an impact on the outcome on the next steps in the reporting process of (suspected) child abuse, because, as was shown by the advisee evaluation, it added to, changed or confirmed the former conclusion of the advisee.

For peer review only - http://bmjopen.bmj.com/site/about/guidelines.xhtml

Teamwork in child abuse diagnostics in itself is not a novel development. In most countries hospitals are required to have some form of a hospital-based multidisciplinary team, and these have proven their value over time [9-11]. Besides these hospital based teams many countries, as well as The Netherlands, have initiated regional or citywide rape and sexual abuse counselling centres, where in many cases specially trained sexual assault nurse examiners (SANE) and/or child abuse paediatricians work [12-14]. These centres play an important role in the diagnosis of sexual child abuse and the care for the victims thereof. The specific aspect of DECCA is the combination of both paediatric and forensic knowledge, outside the field of justice, continuously available on a nationwide scale. Each speciality brings their own expertise. The paediatrician's main focus is on the medical differential diagnosis, whereas the forensic physician adds an objective assessment of the injury and the potential causative mechanism. In addition, the use of Bayes' theorem adds to the perspective of the advisee. This combination of expertise and method yields an integral analysis of the patient and his/her injury from two different viewpoints leading to an objective and evidence based analysis of suspected child abuse, with reference to relevant literature. To our knowledge this makes DECCA unique in the world.

It has been shown, that expert consultation has added value to patient care in cases of suspected child abuse [10 15 16]. However, Linberg et al. showed that even between child abuse paediatricians there was a significant variability in assessing the likelihood of child abuse [17]. They concluded that their data supported the use of peer-review or multidisciplinary teams. The weekly telephone conference, in which all DECCA cases are discussed in-depth, clearly addresses this issue. Not only does this provide a unique opportunity to share knowledge

For peer review only - http://bmjopen.bmj.com/site/about/guidelines.xhtml

and learn from one another, but more importantly, we strongly believe that our approach increases the quality and consistency of the advice given to the referrer.

A limitation of this study is that we could not do a health economics analysis. Long term follow-up of (a subset of) cases will be extremely difficult especially since we register our cases anonymously. With the introduction of the General Data Protection Regulation (GDPR), follow-up would only be possible with consent from both caregivers/parents/legal guardians. This would almost certainly introduce an inclusion bias. No inter-rater reliability assessment is done. However, we restructured our database and as of 2019 we register inconsistencies between the first advice and the joint assessment during the weekly peer-review. This will eventually give us insight in potential shortcomings in our DECCA advice. In addition, the advisee evaluation has been restructured where advisees are requested to fill out a short questionnaire after each consultation. These two procedural changes will form the basis for a new quality assessment.

A major difficulty of diagnosing child abuse lays in one of the principal precepts of bioethics we teach all our medical students: 'primum non nocere'. In cases of potential child abuse the clinician must weigh the risk of underreporting versus overreporting, as both can have serious consequences for the child and its caregivers. As a result it is well known that, even in countries with mandated reporting, there is a significant level of underreporting of child abuse and neglect. One of the explanations for underreporting is reported to be a lack of knowledge leading to uncertainty of the diagnosis of child abuse [18-20]. It could be argued that if expertise centres like DECCA are more widely available this could lead to an increase in

For peer review only - http://bmjopen.bmj.com/site/about/guidelines.xhtml

diagnosing and reporting child abuse, but also in rejecting the diagnosis child abuse when not justified in an earlier phase of the diagnostic process.

The data we present has a significant drawback. As DECCA acts in the first or second step of the guideline as published by the Royal Dutch Medical Association and mainly focusses on findings during the physical examination, there is a distinct referral bias. As a result, cases where physical or sexual child abuse are suspected are overrepresented in our study population in comparison to other child abuse studies. The role of a national paediatric forensic medical expertise centre will in most cases be limited to these two types of child abuse, where physical injury and physical signs are most evident. For cases in which neglect or deprivation play an important role, without signs of physical injury, a face-to-face clinical consultation with a paediatrician and/ or child psychologist with expertise in this field remains the best solution. The strong involvement of three paediatric university hospitals guarantees that in forthcoming cases this service can also be offered to referring health care professionals.

The main challenge DECCA has faced was how to get its work financed. As DECCA works with anonymous cases it therefore cannot bill the patient's insurance company. A grant from two Dutch charity funds, Stichting Kinderpostzegels and Nationale Postcode Loterij, made it possible to start DECCA in 2014 and paid for the running costs for the first year. Currently the Dutch Ministry of Health, Welfare and Sport fully finances our work on a yearly basis. There are no studies into the economic added value of services like DECCA. However, a study into the value of telephone consultations of paediatric subspecialists to primary care physicians was shown to be cost-effective as a result of reduced use of costly services and

For peer review only - http://bmjopen.bmj.com/site/about/guidelines.xhtml

reported improvements in quality of care [21]. We firmly believe that DECCA has a similar impact on patient care and being the sole provider of this care in the Netherlands, DECCA should be financed on a long-term project basis. This financial issue will play a role in other countries as well and could limit the implementation of similar models elsewhere.

Conclusion

Our data shows a rising number of advisory requests and satisfied advisees. We therefore conclude that the Dutch Expertise Centre for Child Abuse seems to be a valuable addition to the Dutch system of child protection.

By virtue of its virtual centralised design this model could, in an adapted form, be established in other countries as well. We are convinced that the combination of experts in the field of paediatrics and forensic medicine including working with Bayes' theorem, and working together with affiliated (sub-)specialties, enables to provide available, evidence-based paediatric forensic medical expertise.

For peer review only - http://bmjopen.bmj.com/site/about/guidelines.xhtml

Table 1. Overview of advisees to DECCA (n=761).

Specialty	N	% of all 761 cases
Paediatrician	404	53.1
Child safeguarding doctors	167	21.9
General practitioner	20	2.6
Youth health care doctor	12	1.6
Emergency Room doctor	9	1.2
Youth care worker	7	0.9
Others (like surgeons, general practitioners or paediatricians in training, psychiatrist, dentist, forensic nurses at ER, nurse practitioners)	142	18.7

For peer review only

For peer review only - http://bmjopen.bmj.com/site/about/guidelines.xhtml

Table 2.

Subspecialties involved in DECCA (n=761).

Subspecialty	N[1]	% of all 761 cases
Paediatric radiology	296	38.9
Paediatric dermatology	31	4.1
Paediatric ophthalmology	22	2.9
Paediatric neurology	12	1.6
Paediatric haematology	7	0.9
Social work	7	0.9
Paediatric surgery	3	0.4
Others (like paediatric urology, ear-nose-throat doctor, paediatric immunology)	15	2.0

[1] The total does not sum up to 761 as not for every case consultant of a subspecialty is needed and several subspecialties can be consulted in one single case.

For peer review only

For peer review only - http://bmjopen.bmj.com/site/about/guidelines.xhtml

Table 3. Findings or red flags leading to a request for DECCA advice

Finding or red flag	N[1]
Injury/ skin lesion	592
Clinical history not in keeping with findings	145
Presence of risk factors in family	70
Physical symptoms	71
Inconsistent clinical history	47
Lesion/trauma not in keeping with child's age	40
Delay in presentation	39
Previous trauma	33
Child admits being a victim of abuse	26
Behavioural symptoms child	19
Caregiver admits to child abuse	17
Improper hygiene child	9
Inadequate interaction child – caregiver	8
Other child in family discloses about child abuse	8
Findings at additional investigations (urine- or bloodtest)	2

[1] The total does not sum up to 761 as several findings or red flags can be present in one single case and several findings can be mentioned in one single case.

For peer review only

For peer review only - http://bmjopen.bmj.com/site/about/guidelines.xhtml

Table 4. Four most common injuries in advisory requests

Most common mentioned type of Injury	N[1]
Bruises	264
Fractures other than skull fracture	166
Skull fracture	69
Brain damage	64

[1] The total does not sum up to 761 as several findings or red flags can be present in one single case and not all injuries are being listed here.

For peer review only

Table 5. Outcome of DECCA evaluation (N=753, 8 missing)

Outcome of DECCA evaluation	Number	% of 753 cases
Almost certainly no child abuse	84	11.1
Child abuse unlikely	185	24.6
Child abuse possible	211	28.0
Child abuse likely	114	15.1
Almost certainly child abuse	69	9.2
Unclear, more investigation needed	90	12.0

For peer review only

For peer review only - http://bmjopen.bmj.com/site/about/guidelines.xhtml

Legend to figures

Figure 1. Stepwise approach of managing child abuse as guideline as published by the Royal Dutch Medical Association [5].

Figure 2. Number of DECCA advisory requests from 2015 to 2018.

Figure 3. Geographic distribution of the advisee to the Dutch Expertise Centre for Child Abuse (N= 740, 21 missing).

For peer review only

For peer review only - http://bmjopen.bmj.com/site/about/guidelines.xhtml

Contributors' Statement
R.R. van Rijn collected data, carried out the initial analyses, and drafted the initial and revised manuscript, and reviewed and revised the manuscript.
M.J. Affourtit designed the data collection instruments and critically reviewed the manuscript.
W.A. Karst designed the data collection instruments and critically reviewed the manuscript.
M. Kamphuis carried out the secondary analyses, and drafted the revised manuscript, and critically reviewed and revised the manuscript.
L.C. de Bock collected data, carried out the secondary analyses and critically reviewed the manuscript
E. van de Putte designed the data collection instruments, and coordinated and supervised data collection, and critically reviewed the manuscript.

All authors approved the final manuscript as submitted and agree to be accountable for all aspects of the work.

All collaborators have read the final manuscript and agree to their name being added to the list of collaborators.

Conflict of Interest:
The authors have no conflicts of interest to disclose.

Funding Source: The Dutch Expertise Centre for Child Abuse is supported by grants from Stichting Kinderpostzegels, Nationale Postcode Loterij, Janivo Stichting, SOD & Fie van der Hoop fonds and governmental support from the Ministry of Health, Welfare and Sport.

Financial Disclosure:
All authors are (part-time or full) employees the Dutch Expertise Centre for Child Abuse.

Data sharing statement
The data, in Dutch, that support the findings of this study are available from the Dutch Expertise Centre for Child Abuse but restrictions apply to the availability of these data, and so are not publicly available. Data are, however, available from the authors upon reasonable request and with permission of DECCA.

For peer review only - http://bmjopen.bmj.com/site/about/guidelines.xhtml

References

1. Felitti VJ, Anda RF, Nordenberg D, et al. Relationship of childhood abuse and household dysfunction to many of the leading causes of death in adults. The Adverse Childhood Experiences (ACE) Study. *Am J PrevMed* 1998;14(4):245-58.

2. Gilbert R, Widom CS, Browne K, et al. Burden and consequences of child maltreatment in high-income countries. *Lancet* 2009;373(9657):68-81. doi: 10.1016/s0140-6736(08)61706-7 [published Online First: 2008/12/06]

3. Irazuzta JE, McJunkin JE, Danadian K, et al. Outcome and cost of child abuse. *Child Abuse Negl* 1997;21(8):751-57.

4. Gelles RJ, Perlman S. Estimated Annual Cost of Child Abuse and Neglect. Chicago IL: Prevent Child Abuse America,, 2012.

5. Royal Dutch Medical Association. KNMG-meldcode kindermishandeling en huiselijk geweld 2018 [Available from: https://www.knmg.nl/advies-richtlijnen/knmg-publicaties/meldcode-kindermishandeling-en-huiselijk-geweld-1.htm.

6. WHO Regional Office for Europe. European report on preventing child maltreatment. In: Sethi D, Bellis M, Hughes K, et al., eds., 2013.

7. Kindermishandeling LEC. [Available from: www.leck.nu accessed 5-10-2017.

8. European Parliament. 2018 reform of EU data protection rules 2018 [Available from: https://ec.europa.eu/commission/priorities/justice-and-fundamental-rights/data-protection/2018-reform-eu-data-protection-rules_en accessed 10-4-2019.

9. Brink FW, Thackeray JD, Bridge JA, et al. Child advocacy center multidisciplinary team decision and its association to child protective services outcomes. *Child Abuse Negl*

For peer review only - http://bmjopen.bmj.com/site/about/guidelines.xhtml

2015;46:174-81. doi: 10.1016/j.chiabu.2015.04.011 [published Online First: 2015/05/11]

10. Draus JM, Jr. A Multidisciplinary Child Protection Team Improves the Care of Nonaccidental Trauma Patients. *The American surgeon* 2017;83(5):477-81. [published Online First: 2017/05/26]

11. Wallace GH, Makoroff KL, Malott HA, et al. Hospital-based multidisciplinary teams can prevent unnecessary child abuse reports and out-of-home placements. *Child Abuse Negl* 2007;31(6):623-9. doi: 10.1016/j.chiabu.2006.12.010 [published Online First: 2007/06/26]

12. Bechtel K, Ryan E, Gallagher D. Impact of sexual assault nurse examiners on the evaluation of sexual assault in a pediatric emergency department. *Pediatric emergency care* 2008;24(7):442-7. doi: 10.1097/PEC.0b013e31817de11d [published Online First: 2008/06/27]

13. Adams JA, Starling SP, Frasier LD, et al. Diagnostic accuracy in child sexual abuse medical evaluation: role of experience, training, and expert case review. *Child Abuse Negl* 2012;36(5):383-92. doi: 10.1016/j.chiabu.2012.01.004 [published Online First: 2012/05/29]

14. Hornor G, Thackeray J, Scribano P, et al. Pediatric sexual assault nurse examiner care: trace forensic evidence, ano-genital injury, and judicial outcomes. *Journal of forensic nursing* 2012;8(3):105-11. doi: 10.1111/j.1939-3938.2011.01131.x [published Online First: 2012/08/29]

15. McGuire L, Martin KD, Leventhal JM. Child abuse consultations initiated by child protective services: the role of expert opinions. *Academic pediatrics* 2011;11(6):467-73. doi: 10.1016/j.acap.2011.06.005 [published Online First: 2011/08/09]

For peer review only

16. Teeuw AH, Sieswerda-Hoogendoorn T, Aaftink D, et al. Assessments carried out by a child abuse and neglect team in an Amsterdam teaching hospital led to interventions in most of the reported cases. *Acta paediatrica (Oslo, Norway : 1992)* 2017;106(7):1118-27. doi: 10.1111/apa.13735 [published Online First: 2016/12/31]

17. Lindberg DM, Lindsell CJ, Shapiro RA. Variability in expert assessments of child physical abuse likelihood. *Pediatrics* 2008;121(4):e945-53. doi: 10.1542/peds.2007-2485 [published Online First: 2008/04/03]

18. Sege R, Flaherty E, Jones R, et al. To report or not to report: examination of the initial primary care management of suspicious childhood injuries. *Academic pediatrics* 2011;11(6):460-6. doi: 10.1016/j.acap.2011.08.005 [published Online First: 2011/10/15]

19. Talsma M, Bengtsson Bostrom K, Ostberg AL. Facing suspected child abuse--what keeps Swedish general practitioners from reporting to child protective services? *Scandinavian journal of primary health care* 2015;33(1):21-6. doi: 10.3109/02813432.2015.1001941 [published Online First: 2015/02/14]

20. Vulliamy AP, Sullivan R. Reporting child abuse: pediatricians' experiences with the child protection system. *Child Abuse Negl* 2000;24(11):1461-70. [published Online First: 2000/12/29]

21. Wegner SE, Humble CG, Feaganes J, et al. Estimated savings from paid telephone consultations between subspecialists and primary care physicians. *Pediatrics* 2008;122(6):e1136-40. doi: 10.1542/peds.2008-0432 [published Online First: 2008/12/03]

For peer review only - http://bmjopen.bmj.com/site/about/guidelines.xhtml

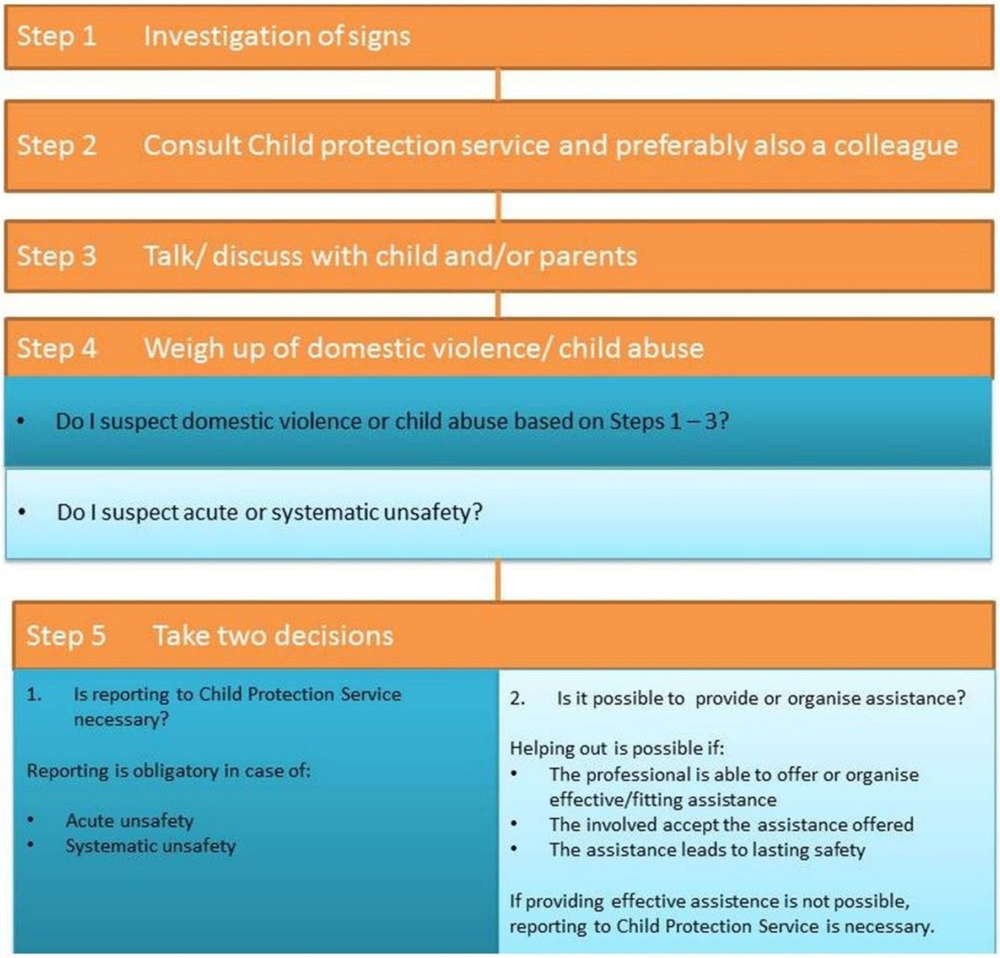

Figure 1. Stepwise approach of managing child abuse as guideline as published by the Royal Dutch Medical Association

90x90mm (300 x 300 DPI)

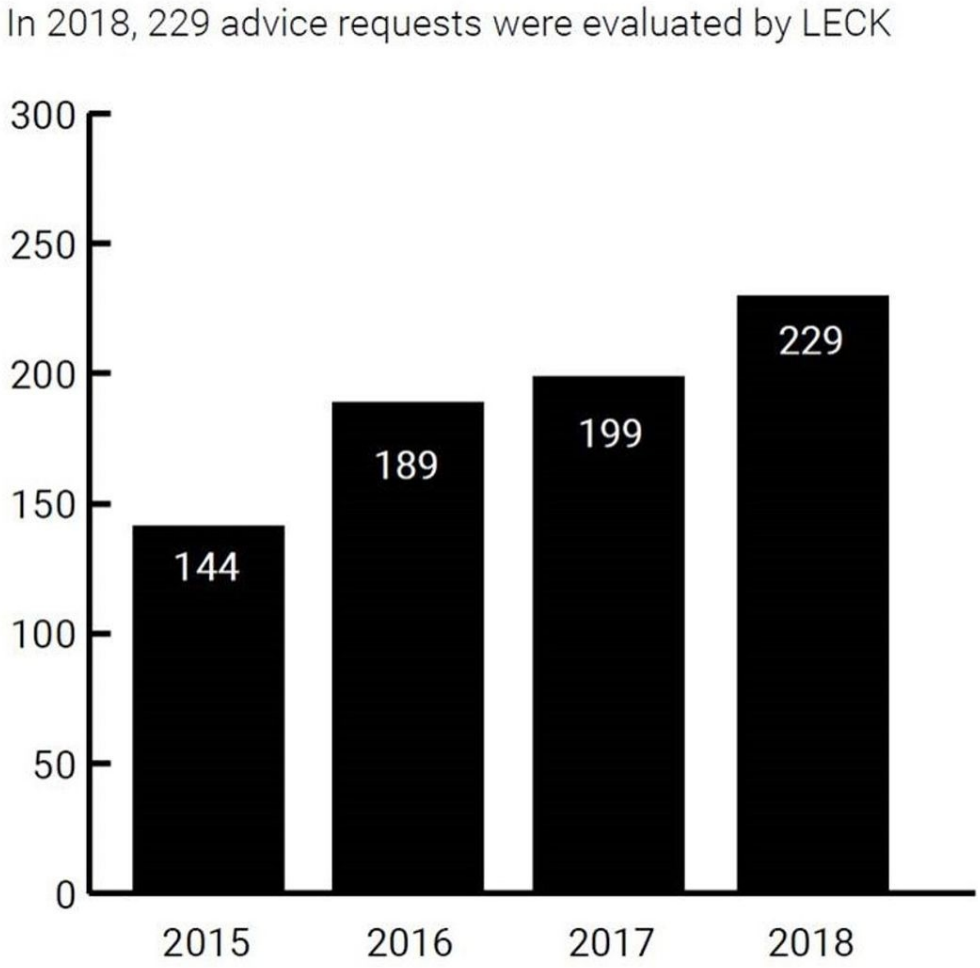

Figure 2.Number of DECCA advisory requests from 2015 to 2018.

90x90mm (300 x 300 DPI)

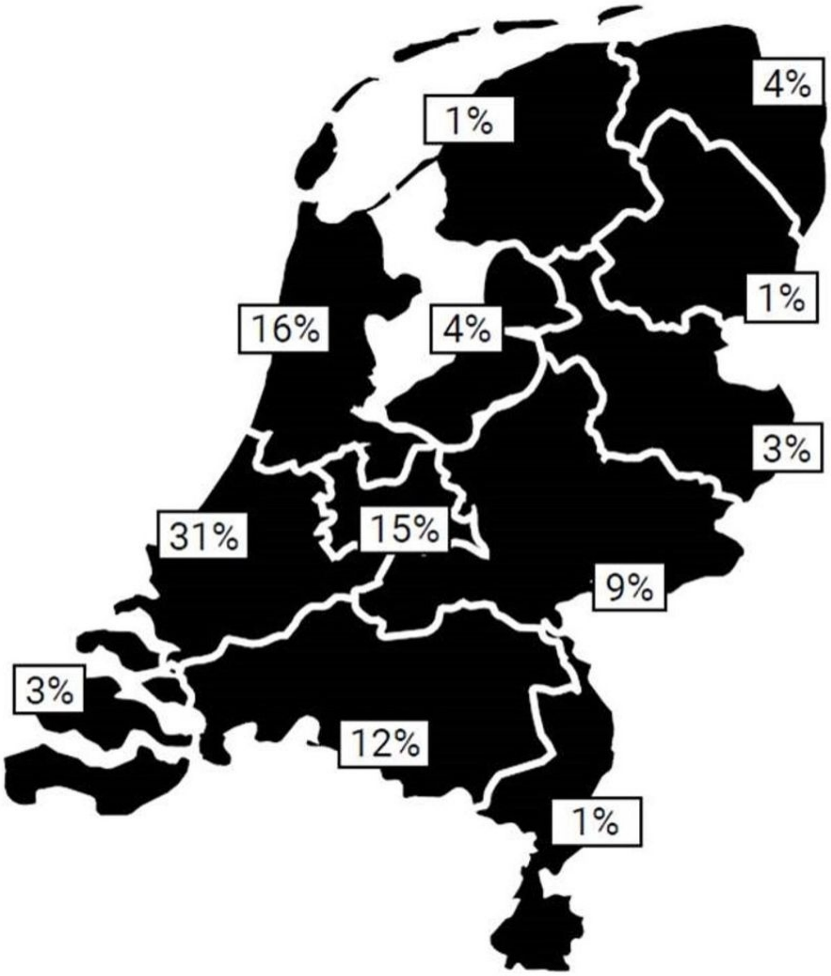

Figure 3. Geographic distribution of the advisee to the Dutch Expertise Centre for Child Abuse (N= 740, 21 missing).

90x90mm (300 x 300 DPI)

STROBE Statement - The start of the Dutch Expertise Centre for Child Abuse; a descriptive study of the implementation and data of the first 4 years

	Item	Recommendation	Page
**Title and abstract**	1	(*a*) Indicate the study's design with a commonly used term in the title or the abstract	Title page
		(*b*) Provide in the abstract an informative and balanced summary of what was done and what was found	3
**Introduction**			
Background/rationale	2	Explain the scientific background and rationale for the investigation being reported	6
Objectives	3	State specific objectives, including any prespecified hypotheses	7
**Methods**			
Study design	4	Present key elements of study design early in the paper	10
Setting	5	Describe the setting, locations, and relevant dates, including periods of recruitment, exposure, follow-up, and data collection	10
Participants	6	(*a*) *Cohort study*—Give the eligibility criteria, and the sources and methods of selection of participants. Describe methods of follow-up<br>*Case-control study*—Give the eligibility criteria, and the sources and methods of case ascertainment and control selection. Give the rationale for the choice of cases and controls<br>*Cross-sectional study*—Give the eligibility criteria, and the sources and methods of selection of participants	10
		(*b*) *Cohort study*—For matched studies, give matching criteria and number of exposed and unexposed<br>*Case-control study*—For matched studies, give matching criteria and the number of controls per case	N/A
Variables	7	Clearly define all outcomes, exposures, predictors, potential confounders, and effect modifiers. Give diagnostic criteria, if applicable	N/A
Data sources/ measurement	8	For each variable of interest, give sources of data and details of methods of assessment (measurement). Describe comparability of assessment methods if there is more than one group	N/A
Bias	9	Describe any efforts to address potential sources of bias	N/A
Study size	10	Explain how the study size was arrived at	N/A
Quantitative variables	11	Explain how quantitative variables were handled in the analyses. If applicable, describe which groupings were chosen and why	N/A
Statistical methods	12	(*a*) Describe all statistical methods, including those used to control for confounding	11
		(*b*) Describe any methods used to examine subgroups and interactions	N/A
		(*c*) Explain how missing data were addressed	N/A
		(*d*) *Cohort study*—If applicable, explain how loss to follow-up was addressed<br>*Case-control study*—If applicable, explain how matching of cases and controls was addressed	N/A

For peer review only

For peer review only - http://bmjopen.bmj.com/site/about/guidelines.xhtml

		Cross-sectional study—If applicable, describe analytical methods taking account of sampling strategy	
		(*e*) Describe any sensitivity analyses	N/A
Results			
Participants	13*	(a) Report numbers of individuals at each stage of study—eg numbers potentially eligible, examined for eligibility, confirmed eligible, included in the study, completing follow-up, and analysed	N/A
		(b) Give reasons for non-participation at each stage	N/A
		(c) Consider use of a flow diagram	N/A
Descriptive data	14*	(a) Give characteristics of study participants (eg demographic, clinical, social) and information on exposures and potential confounders	10
		(b) Indicate number of participants with missing data for each variable of interest	N/A
		(c) *Cohort study*—Summarise follow-up time (eg, average and total amount)	N/A
Outcome data	15*	*Cohort study*—Report numbers of outcome events or summary measures over time	N/A
		Case-control study—Report numbers in each exposure category, or summary measures of exposure	N/A
		Cross-sectional study—Report numbers of outcome events or summary measures	N/A
Main results	16	(*a*) Give unadjusted estimates and, if applicable, confounder-adjusted estimates and their precision (eg, 95% confidence interval). Make clear which confounders were adjusted for and why they were included	N/A
		(*b*) Report category boundaries when continuous variables were categorized	N/A
		(*c*) If relevant, consider translating estimates of relative risk into absolute risk for a meaningful time period	N/A
Other analyses	17	Report other analyses done—eg analyses of subgroups and interactions, and sensitivity analyses	N/A
**Discussion**			
Key results	18	Summarise key results with reference to study objectives	13
Limitations	19	Discuss limitations of the study, taking into account sources of potential bias or imprecision. Discuss both direction and magnitude of any potential bias	15
Interpretation	20	Give a cautious overall interpretation of results considering objectives, limitations, multiplicity of analyses, results from similar studies, and other relevant evidence	13
Generalisability	21	Discuss the generalisability (external validity) of the study results	16
**Other information**			
Funding	22	Give the source of funding and the role of the funders for the present study and, if applicable, for the original study on which the present article is based	16

For peer review only

BMJ Open

Implementation of the Dutch Expertise Centre for Child Abuse: descriptive data from the first four years

Journal:	*BMJ Open*
Manuscript ID	bmjopen-2019-031008.R2
Article Type:	Research
Date Submitted by the Author:	05-Jul-2019
Complete List of Authors:	van Rijn, Rick; Emma Children's hospital, Academic Medical Center, radiology; Nederlands Forensisch Instituut, Forensic Medicine<br>Affourtit, Marjo; Erasmus MC Sophia Children's Hospital, Pediatrics<br>Karst, Wouter; Nederlands Forensisch Instituut, Forensic Medicine<br>Kamphuis, Mascha; Dutch Expertise Center for Child Abuse<br>de Bock, Leonie; Dutch Expertise Center for Child Abuse<br>van de Putte, Elise; University Medical Center Utrecht, General Paediatrics
**Primary Subject Heading**:	Paediatrics
Secondary Subject Heading:	Paediatrics
Keywords:	Child protection < PAEDIATRICS, Child Abuse, FORENSIC MEDICINE

SCHOLARONE™
Manuscripts

Implementation of the Dutch Expertise Centre for Child Abuse: descriptive data from the first four years

R.R. van Rijn[1,2#,3], M.J. Affourtit[3,4], W.A. Karst[2,3], M. Kamphuis[3], L.C. de Bock[3], E. van de Putte[3,5], on behalf of the Dutch Expertise Centre for Child Abuse Study Group

Department of Radiology[1], Emma Children's Hospital - Amsterdam UMC, University of Amsterdam, Amsterdam, the Netherlands

Department of Forensic Medicine[2], Netherlands Forensic Institute, the Hague, the Netherlands

Dutch Expertise Center for Child Abuse[3], Utrecht, the Netherlands

Department of Paediatrics[4], Sophia Children's Hospital – ErasmusMC, Rotterdam, the Netherlands

Department of Paediatrics[5], Wilhelmina Children's Hospital – UMC Utrecht, Utrecht, the Netherlands

For peer review only

[#] Address for correspondence:
Prof. Rick R. van Rijn, MD. PhD.
Department of Radiology and Nuclear Medicine
Emma Children's Hospital – Amsterdam UMC, University of Amsterdam
Meibergdreef 9
1105 AZ Amsterdam Zuid-Oost
the Netherlands
Tel: 31-20-5669111
E-mail: r.r.vanrijn@amc.uva.nl

For peer review only - http://bmjopen.bmj.com/site/about/guidelines.xhtml

Collaborators of the Dutch Expertise Centre for Child Abuse Study Group are:

- Department of Forensic Medicine, Netherlands Forensic Institute, the Hague, the Netherlands. Collaborators:
  - R.A.C. Bilo, MD.
  - Mirjam Kruijsen-Jaarsma, MD.
  - H.G.T. Nijs, MD. PhD.
  - H.C. Terlingen, MD.
  - S. de Vries, MD.
  - N.L. van Woerden, MD. PhD.

- Department of Paediatrics, Emma Children's Hospital - Amsterdam UMC, University of Amsterdam, the Netherlands, Collaborators:
  - L. van der Berg, MD.
  - A.C.M. van Bellegem, MD.
  - M. Bouman, MD.
  - M.H. Loos, MD.
  - A.S. Smeijers, MD.
  - A.H. Teeuw, MD. PhD.

- Department of Paediatrics, Sophia Children's Hospital – ErasmusMC, Rotterdam, the Netherlands. Collaborators:
  - P.J. Puiman, MD. PhD.

- Department of Paediatrics, Wilhelmina Children's Hospital – UMC Utrecht, Utrecht, the Netherlands. Collaborators:
  - F.M.C. van Berkestijn, MD.
  - F. Kamberg
  - S.L. Nijhof, MD. PhD.
  - J.M. Ruskamp, MD. PhD.
  - I.M.B. Russel-Kampschoer, MD.
  - M. Schouten, MD. PhD.
  - K. Sijstermans, MD. PhD.
  - S.A.A. Wolt-Plompen, MD.

Keywords
Child protection
Child Abuse
Forensic Medicine

Wordcount
3843

Abbreviations:
DECCA = Dutch Expertise Centre for Child Abuse

For peer review only - http://bmjopen.bmj.com/site/about/guidelines.xhtml

For peer review only

For peer review only

Abstract

Objective

Combined paediatric and forensic medical expertise to interpret physical findings is not available in Dutch healthcare facilities. The Dutch Expertise Centre for Child Abuse (DECCA) was founded in the conviction that this combination is essential in assessing potential physical child abuse. DECCA is a collaboration between three paediatric hospitals and the Netherlands Forensic Institute. DECCA works with Bayes' theorem and uses likelihood ratios in their conclusions.

Design

We present the implementation process of DECCA and cross sectional data of the first four years.

Participants

Between December 14th 2014 and December 31st 2018 a total of 761 advisory requests were referred, all of which were included in this study. An advisee evaluation over the year 2015 was performed using a self-constructed survey to gain insight in the first experiences with DECCA.

Results

761 cases were included, 381 (50.1%) boys and 361 (47.4%) girls (19 cases (2.5%) sex undisclosed). Median age was 1.5 years (range 1 day – 20 year).

Paediatricians (53.1%) and child safeguarding doctors (21.9%) most frequently contacted DECCA.

Two most common reasons for referral were presence of injury/skin lesions (n=592) and clinical history inconsistent with findings (n=145). The most common injuries were bruises (264) and non-skull fractures (166).

For peer review only - http://bmjopen.bmj.com/site/about/guidelines.xhtml

Outcome of DECCA evaluation was almost certainly no or improbable child abuse in 35.7%; child abuse likely or almost certain in 24.3%; and unclear in 12%.

The advisee evaluations (response rate 50%) showed that 93% experienced added value and that 100% were (very) satisfied with the advice.

Conclusion

Data show growing interest in the expertise of DECCA through the years. DECCA seems to be a valuable addition to Dutch child protection, since advisee value the service and outcome of DECCA evaluations. In almost half of the cases DECCA concluded that child abuse could not be substantiated.

For peer review only

Strengths and limitations of this study

- After four years of advisory requests, we have collected a large number of cases to show solid results. With the growing number of advisory requests and satisfied advisees, we were able to show that organising and providing round-the-clock, expert based independent high quality combined paediatric and forensic medical expertise on a national level is feasible.
- During the initial discussion between the paediatrician, who has spoken with the advisee, and the forensic physician, the latter is blinded with respect to the social history and other risk factors. This way we aim to avoid cognitive bias as much as possible.
- As the Dutch Expertise Centre for Child Abuse works with anonymous advice follow-up of the individual cases is not possible. Therefore a health economics analysis, although important, is impossible.
- The results of the advisee evaluations could be biased, because possibly only satisfied advisees responded to the survey.
- An update of the advisee evaluations is needed and this has been implemented as a routine part of our protocol since 1-1-2019.

For peer review only

For peer review only - http://bmjopen.bmj.com/site/about/guidelines.xhtml

Introduction

Child abuse is a world-wide ubiquitous problem leading to both short term and long term negative outcomes in health and social well-being of the victims [1 2]. It has a detrimental impact on the development of children, with an impact on the individual throughout life. The seminal study by Fellitti et al. has shown that there is an increase of disease and early death in adulthood in child abuse survivors [1]. Besides the direct impact on the victim, it has been shown to have a significant cost to society [3 4].

Both from an individual as well as a societal point of view it is important to prevent and detect child abuse as early as possible and intervene when child abuse is suspected in order to prevent further abuse and to improve the outcome of the individual victim and his/her social network.

In the Netherlands, in contrast to other European countries and the United States of America, physicians are only mandated to report child abuse in specific cases according to the guideline Domestic violence and child abuse as published by the Royal Dutch Medical Association (figure 1) [5]. This guideline dictates five steps, of which the first step is to collect information about the patient in question and assess the potential for the presence of child abuse. If the attending physician thinks that child abuse might be present he/she is required to ask advice from or report to so-called child safeguarding doctors (step 2). Child safeguarding doctors work regionally within a national system in which all cases of possible child abuse are reported and investigated and they can, in collaboration with a wide variety of health care support teams, instigate interventions.

However, in order to substantiate potential abuse as well as reject this diagnosis in the first step of the diagnostic pathway, specifically concerning injuries on physical examination and questions concerning physical signs and symptoms, in-depth knowledge of both paediatric as

For peer review only - http://bmjopen.bmj.com/site/about/guidelines.xhtml

well as forensic medicine is required. We will refer to this combination of expertise as combined paediatric forensic medical expertise. In addition, other relevant sub-specialties and affiliated specialties such as radiology, will add to this knowledge. This adheres to the recommendation of the WHO Regional Office for Europe report on the prevention of child maltreatment, to use a multidisciplinary approach of cases that use reliable and valid investigative methods (2013) [6]. In the Netherlands, as in most countries, this broad combined range of specialties is not available in hospitals or health care facilities. Given this fact and the necessity for 24/7 support for health care professionals confronted with a potential case of child abuse, we founded the 'Landelijk Expertise Centrum Kindermishandeling' (Dutch Expertise Centre for Child Abuse (DECCA)) in 2014 [7]. DECCA can be consulted in step 1, step 2, and step 5 of the national guideline Domestic violence and child abuse [5]. Child death reviews are not part of the objectives of DECCA. In this publication we present the organisation and methods of the DECCA and our initial experiences in the first four years.

Organisation of the DECCA

Advice and consultations are performed by DECCA-paediatricians and DECCA-forensic physicians. The DECCA is a tax exempted foundation initiated by three university paediatric hospitals (Emma Children's Hospital – Amsterdam UMC – University of Amsterdam, Sophia Children's Hospital – ErasmusMC Rotterdam, and Wilhelmina Children's Hospital – UMC Utrecht) and the department of forensic medicine of the Netherlands Forensic Institute, The Hague. The DECCA as such is a virtual organisation: it has no buildings. DECCA is supervised by a board (consisting of a physician from each centre and a treasurer), a central coordinator, a secretary and since 2018 a medical director. In each centre one of the

For peer review only - http://bmjopen.bmj.com/site/about/guidelines.xhtml

involved physicians is in the lead and these four team leaders meet on a semi-annual basis with the board of DECCA to discuss points for improvement and future developments. In addition, an advisory board and advisors meet up with the board two to three times a year.

Method of DECCA

The DECCA is available for health care professionals seven days a week, day and night, by a national telephone number which is staffed by a paediatrician with expertise and experience in child abuse diagnostics (an additional two and half years of education). At the same time a forensic physician, with expertise and experience in child abuse evaluation is on call. Specific quality criteria for the physicians working at DECCA are described and are maintained by the board and medical director of DECCA.

Advisees of the DECCA are mainly medical doctors who ask for a one-off advice (by telephone or email) or refer for consultation. Besides medical information, photographs and radiography can be provided for review. Focus is mainly on physical injury and questions concerning physical signs and symptoms. For all cases where advice is requested DECCA records the patient’s data anonymously. Because of this DECCA has a low threshold for physicians, as in the Netherlands anonymous advice can be asked without permission from parents or caregivers.

In all cases the advisee will get a combined advice from both a paediatrician and a forensic physician. During the initial discussion between the paediatrician, who has spoken with the advisee, and the forensic physician, the latter is blinded with respect to the social history and other risk factors. This way we aim to avoid cognitive bias as much as possible. At first a conclusion is formed using a likelihood ratio (, that is the likelihood that the medical findings or injuries would be expected in an abused child compared to a non-abused child). This

For peer review only - http://bmjopen.bmj.com/site/about/guidelines.xhtml

conclusion is substantiated with data from scientific publications. To determine the likelihood of abuse, all other information (apart from the injuries) should be used to estimate a prior probability of abuse. Hence the injuries should not be used to determine the prior probability of abuse, they should only be used to determine the likelihood ratio. Using Bayesian reasoning, the prior odds of abuse can be multiplied by the likelihood ratio that was determined by the forensic physician who was blinded for risk factors of abuse. This way, cognitive bias will be avoided as much as possible. As the information needed to determine the prior probability of abuse might be incomplete, subjective, unknown or outside the field of expertise of medical doctors, a correct (posterior) probability of abuse cannot be given by DECCA. If a probability is needed to determine further action or evaluation, the prevalence of abuse could be used as prior probability. If DECCA provides prevalence data in specific situations, it is always stated that using those data to determine the probability of abuse might be the best solution, but also might be incorrect.

The search for evidence and the discussion between the paediatrician and the forensic physician continues until they agree. In case of different opinions or lack of evidence, additional advice is obtained from other experts both nationally as well as internationally. In all cases in which DECCA is asked for advice, the advisee will receive a letter stating the anonymous information provided by the advisee, the conclusions reached by DECCA and if needed the advice for further assessment or follow-up. It is specifically stated that this letter should be included in the patient's medical record. All cases are recorded in a secure on-line data base (Castor EDC®, CIWIT B.V., Amsterdam, the Netherlands) to which all DECCA professionals have password protected access to, including two-factor authentication. The database conforms to the guidelines of good clinical practice and the General Data Protection Regulation [8]. Advice will be given anonymously and personal information of the

For peer review only - http://bmjopen.bmj.com/site/about/guidelines.xhtml

child or the family is not added to our data base. Name and email address of the advisee is included in the data base, in order to be able to send the letter of advice to the advisee.

A DECCA case-review telephone conference in which all centres are engaged and at least two senior paediatricians and a forensic physician must be present is held weekly. During this telephone conference all cases of the past week are discussed anonymously. In this discussion the paediatrician who initially handled the case presents it to the participating DECCA physicians and the advice given is discussed, including the written report. Based on the group discussion the advice can be altered. In case of an updated or altered advice the referring health care professional is contacted to discuss this update. The DECCA is not involved in further treatment or follow-up of the cases.

In those cases in which additional specific expertise for physical examination or additional investigations is required, DECCA can advise referral of the patient to a paediatric centre with expertise on child abuse (including one of the three centres collaborating in the DECCA) for out-patient follow-up and evaluation. For example, this could be the case in investigation of signs of sexual abuse. On average 35 cases per year are referred to one of the three academic paediatric hospitals of DECCA for face-to-face consultation. These cases are part of academic patient care, hence not the main focus of DECCA, and are therefore not presented in this overview.

Methods: Data collection and statistical analyses

Between December 14th 2014 and December 31st 2018 a total of 761 advisory requests were recorded by the DECCA paediatricians in the DECCA database.

Additionally, an evaluation of the experiences of advisees was performed. This survey was specifically developed for this study and is available (in Dutch) upon request. All advisees

For peer review only - http://bmjopen.bmj.com/site/about/guidelines.xhtml

that consulted DECCA from December 2014 until December 2015 received an online survey consisting of fifteen questions about their advisory request. This related to how they experienced the added value of DECCA, if they were satisfied with the advice as such, and how DECCA could improve their service. This evaluation was done from May 2015 to January 2016. During this time three reminders were send. In total 122 advisees were asked to participate anonymously in an on-line questionnaire sent directly after they had received advice.

Statistical analysis was performed using IBM SPSS Statistics for Windows, Version 25. (Armonk, NY: IBM Corp.). Analyses involved the processing of frequencies or cross tables.

Patient and Public Involvement

As DECCA does not record patient identifiers, patients and/or their parents could not be included in this descriptive study of the first four years of DECCA.

Ethical approval

The internal review board of the University Medical Centre Utrecht, where DECCA has a postal address, issued a waiver for the documentation of consent and approved the use of an anonymized database.

Results

Advisory requests

All cases of advisory requests recorded in the data base were included in the analyses, this concerns a total of 761. Figure 2 shows the increasing number of requests during the years. There were 381 (50.1%) boys and 361 (47.4%) girls; in 19 cases (2.5%) sex was not disclosed.

For peer review only - http://bmjopen.bmj.com/site/about/guidelines.xhtml

Age was not recorded in 9 cases. The median age of the referred population was 1.5 years (IQR 3.67, range 1 day – 20 years). The oldest case was a 20 year-old patient who, although too old according to the definition of child abuse, was a sibling of a younger child and was therefore referred to DECCA and included in this study. The median age in boys was 1.4 years (IQR 3.06) and in girls this was 2 years (IQR 5.35). The majority of cases was referred from the western provinces of The Netherlands, corresponding to a more densely populated area (figure 3). The cases were referred throughout the week, though mainly during weekdays (91.9%). Cases were referred throughout the day and night, however mostly during office hours (75.9%).

There was a wide variety of health care professionals who consulted the DECCA for advice on cases in which they had findings concerning (suspected) child abuse (table 1). Paediatricians were the most prevalent advisees (53.1%), with child safeguarding doctors coming second. This is shifting over the years towards relatively more requests from child safeguarding doctors (from 21% in 2015 to 26% in 2018) and relatively less from paediatricians (from 59% in 2015 to 52% in 2018) (table 1).

In some cases expertise from other medical specialists was needed, DECCA therefore involved several clinical subspecialties in the advice process besides paediatrics and forensics, primarily paediatric radiology (table 2).

The primary question of the advisee mainly concerned 'Does the observed injury fit the described trauma mechanism?' and 'Could the nature of the injury be ascertained?'. As is common in (suspected) child abuse there were often more than one finding or so-called red flags leading to a request for DECCA advice (table 3). Most common injuries in advisory requests were bruises (264), non-skull fractures (166), skull fractures (69) and brain injury (64) (table 4).

For peer review only

For peer review only - http://bmjopen.bmj.com/site/about/guidelines.xhtml

In addition to answering the specific question of the advisee and when possible, the conclusion of DECCA advice specifically states a level of certainty with respect to child abuse. Table 5 shows the outcome regarding the possibility of child abuse as concluded by DECCA. This was almost certainly no or improbable child abuse in 35.7%; child abuse possible in 28%; and child abuse likely or almost certain in 24.3%. In 12% of cases additional investigations were advised.

Advisee evaluation:

In total 61 out of 122 (50%) advisees responded to the online evaluation questionnaire. Of these respondents 39 (64%) stated that this was the first time they contacted DECCA and the rest (36%) stated that they had previously been in contact with DECCA. In the majority of cases the respondents found that the DECCA advice had either much additional value (N=51 (84%)) or very much additional value (N=8 (13%)). Only two respondents (3%) found that the DECCA advice was of little additional value. The DECCA advice changed the perspective on the case in 33 (54%) of cases. In all cases the respondents were satisfied or very satisfied about the DECCA advise and marked it on average as 8.4 on a scale from 0 to 10 (range from 7 to 10, with 16 respondents scoring 9 and 5 scoring 10).

For peer review only

Discussion

Interest for contacting DECCA is growing nationally, as is shown by the increasing numbers of advisory requests (from 132 in 2015 to 229 in 2018). In the past four years DECCA has shown that organising and providing expert-based and independent combined paediatric and forensic medical expertise on a national level is feasible. From both a care providers' as well as the child's viewpoint DECCA's involvement had an impact on the outcome on the next

For peer review only - http://bmjopen.bmj.com/site/about/guidelines.xhtml

steps in the reporting process of (suspected) child abuse, because, as was shown by the advisee evaluation, it added to, changed or confirmed the former conclusion of the advisee.

Teamwork in child abuse diagnostics in itself is not a novel development. In many countries hospitals have some form of a hospital-based multidisciplinary team, and these have proven their value over time [9-12]. Besides these hospital based teams many countries, as well as The Netherlands, have initiated regional or citywide rape and sexual abuse counselling centres, where in many cases specially trained sexual assault nurse examiners (SANE) and/or child abuse paediatricians work [13-15]. These centres play an important role in the diagnosis of sexual child abuse and the care for the victims thereof. The specific aspect of DECCA is the combination of both paediatric and forensic knowledge, outside the field of justice, continuously available on a nationwide scale. Each speciality brings their own expertise. The paediatrician's main focus is on the medical differential diagnosis, whereas the forensic physician adds an objective assessment of the injury and the potential causative mechanism. In addition, the use of Bayes' theorem adds to the perspective of the advisee. This combination of expertise and method yields an integral analysis of the patient and his/her injury from two different viewpoints leading to an objective and evidence based analysis of suspected child abuse, with reference to relevant literature. To our knowledge this makes DECCA unique in the world.

It has been shown, that expert consultation has added value to patient care in cases of suspected child abuse [10 16 17]. However, Lindberg et al. showed that even between child abuse paediatricians there was a significant variability in assessing the likelihood of child abuse [18]. They concluded that their data supported the use of peer-review or

For peer review only - http://bmjopen.bmj.com/site/about/guidelines.xhtml

multidisciplinary teams. The weekly telephone conference, in which all DECCA cases are discussed in-depth, clearly addresses this issue. Not only does this provide an excellent opportunity to share knowledge and learn from one another, but more importantly, we strongly believe that our approach increases the quality and consistency of the advice given to the referrer.

A limitation of this study is that we could not do a health economics analysis. Long term follow-up of (a subset of) cases will be extremely difficult especially since we register our cases anonymously. With the introduction of the General Data Protection Regulation (GDPR), follow-up would only be possible with consent from both caregivers/parents/legal guardians. This would almost certainly introduce an inclusion bias. No inter-rater reliability assessment is done. However, we restructured our database and as of 2019 we register inconsistencies between the first advice and the joint assessment during the weekly peer-review. This will eventually give us insight in potential shortcomings in our DECCA advice. In addition, the advisee evaluation has been restructured where advisees are requested to fill out a short questionnaire after each consultation. These two procedural changes will form the basis for a new quality assessment.

A major difficulty of diagnosing child abuse lies in one of the principal precepts of bioethics we teach all our medical students: ‘primum non nocere’. In cases of potential child abuse the clinician must weigh the risk of underreporting versus overreporting, as both can have serious consequences for the child and its caregivers. As a result it is well known that, even in countries with mandated reporting, there is a significant level of underreporting of child abuse and neglect. One of the explanations for underreporting is reported to be a lack of

For peer review only - http://bmjopen.bmj.com/site/about/guidelines.xhtml

knowledge leading to uncertainty of the diagnosis of child abuse [19-21]. It could be argued that if expertise centres like DECCA are more widely available this could lead to an increase in diagnosing and reporting child abuse, but also in rejecting the diagnosis child abuse when not justified in an earlier phase of the diagnostic process.

The data we present has a significant drawback. As DECCA acts in the first or second step of the guideline as published by the Royal Dutch Medical Association and mainly focusses on findings during the physical examination, there is a distinct referral bias. As a result, cases where physical or sexual child abuse are suspected are overrepresented in our study population in comparison to other child abuse studies. The role of a national paediatric forensic medical expertise centre will in most cases be limited to these two types of child abuse, where physical injury and physical signs are most evident. For cases in which neglect or deprivation play an important role, without signs of physical injury, a face-to-face clinical consultation with a paediatrician and/ or child psychologist with expertise in this field remains the best solution. The strong involvement of three paediatric university hospitals guarantees that in forthcoming cases this service can also be offered to referring health care professionals.

The main challenge DECCA has faced was how to get its work financed. As DECCA works with anonymous cases it therefore cannot bill the patient's insurance company. A grant from two Dutch charity funds, Stichting Kinderpostzegels and Nationale Postcode Loterij, made it possible to start DECCA in 2014 and paid for the running costs for the first year. Currently the Dutch Ministry of Health, Welfare and Sport fully finances our work on a yearly basis. There are no studies into the economic added value of services like DECCA. However, a study

For peer review only

into the value of telephone consultations of paediatric subspecialists to primary care physicians was shown to be cost-effective as a result of reduced use of costly services and reported improvements in quality of care [22]. We firmly believe that DECCA has a similar impact on patient care and being the sole provider of this care in the Netherlands, DECCA should be financed on a long-term project basis. This financial issue will play a role in other countries as well and could limit the implementation of similar models elsewhere.

Conclusion

Our data shows a rising number of advisory requests and satisfied advisees. We therefore conclude that the Dutch Expertise Centre for Child Abuse seems to be a valuable addition to the Dutch system of child protection.

By virtue of its virtual centralised design this model could, in an adapted form, be established in other countries as well. We are convinced that the combination of experts in the field of paediatrics and forensic medicine including working with Bayes' theorem, and working together with affiliated (sub-)specialties, enables to provide available, evidence-based paediatric forensic medical expertise.

For peer review only

For peer review only - http://bmjopen.bmj.com/site/about/guidelines.xhtml

Table 1. Overview of advisees to DECCA (n=761).

Specialty	N	% of all 761 cases
Paediatrician	404	53.1
Child safeguarding doctors	167	21.9
General practitioner	20	2.6
Youth health care doctor	12	1.6
Emergency Room doctor	9	1.2
Youth care worker	7	0.9
Others (like surgeons, general practitioners or paediatricians in training, psychiatrist, dentist, forensic nurses at ER, nurse practitioners)	142	18.7

For peer review only

For peer review only - http://bmjopen.bmj.com/site/about/guidelines.xhtml

Table 2.

Subspecialties involved in DECCA (n=761).

**Subspecialty**	**N[1]**	**% of all 761 cases**
Paediatric radiology	296	38.9
Paediatric dermatology	31	4.1
Paediatric ophthalmology	22	2.9
Paediatric neurology	12	1.6
Paediatric haematology	7	0.9
Social work	7	0.9
Paediatric surgery	3	0.4
Others (like paediatric urology, ear-nose-throat doctor, paediatric immunology)	15	2.0

[1] The total does not sum up to 761 as not for every case consultant of a subspecialty is needed and several subspecialties can be consulted in one single case.

For peer review only

For peer review only - http://bmjopen.bmj.com/site/about/guidelines.xhtml

Table 3. Findings or red flags leading to a request for DECCA advice

Finding or red flag	N[1]
Injury/ skin lesion	592
Clinical history not in keeping with findings	145
Presence of risk factors in family	70
Physical symptoms	71
Inconsistent clinical history	47
Lesion/trauma not in keeping with child's age	40
Delay in presentation	39
Previous trauma	33
Child admits being a victim of abuse	26
Behavioural symptoms child	19
Caregiver admits to child abuse	17
Improper hygiene child	9
Inadequate interaction child – caregiver	8
Other child in family discloses about child abuse	8
Findings at additional investigations (urine- or bloodtest)	2

[1] The total does not sum up to 761 as several findings or red flags can be present in one single case and several findings can be mentioned in one single case.

For peer review only

Table 4. Four most common injuries in advisory requests

Most common mentioned type of Injury	N[1]
Bruises	264
Non-skull fracture	166
Skull fracture	69
Brain damage	64

[1] The total does not sum up to 761 as several findings or red flags can be present in one single case and not all injuries are being listed here.

For peer review only

For peer review only - http://bmjopen.bmj.com/site/about/guidelines.xhtml

1 **Table 5. Outcome of DECCA evaluation (N=753, 8 missing)**

2

Outcome of DECCA evaluation	Number	% of 753 cases
Almost certainly no child abuse	84	11.1
Child abuse unlikely	185	24.6
Child abuse possible	211	28.0
Child abuse likely	114	15.1
Almost certainly child abuse	69	9.2
Unclear, more investigation needed	90	12.0

3

For peer review only

For peer review only - http://bmjopen.bmj.com/site/about/guidelines.xhtml

Legend to figures

Figure 1. Stepwise approach of managing child abuse as guideline as published by the Royal Dutch Medical Association [5].

Figure 2. Number of DECCA advisory requests from 2015 to 2018.

Figure 3. Geographic distribution of the advisee to the Dutch Expertise Centre for Child Abuse (N= 740, 21 missing).

For peer review only

For peer review only - http://bmjopen.bmj.com/site/about/guidelines.xhtml

Contributors' Statement

R.R. van Rijn collected data, carried out the initial analyses, and drafted the initial and revised manuscript, and reviewed and revised the manuscript.

M.J. Affourtit designed the data collection instruments and critically reviewed the manuscript.

W.A. Karst designed the data collection instruments and critically reviewed the manuscript.

M. Kamphuis carried out the secondary analyses, and drafted the revised manuscript, and critically reviewed and revised the manuscript.

L.C. de Bock collected data, carried out the secondary analyses and critically reviewed the manuscript

E. van de Putte designed the data collection instruments, and coordinated and supervised data collection, and critically reviewed the manuscript.

All authors approved the final manuscript as submitted and agree to be accountable for all aspects of the work.

All collaborators have read the final manuscript and agree to their name being added to the list of collaborators.

Conflict of Interest:

The authors have no conflicts of interest to disclose.

Funding Source: The Dutch Expertise Centre for Child Abuse is supported by grants from Stichting Kinderpostzegels, Nationale Postcode Loterij, Janivo Stichting, SOD & Fie van der Hoop fonds and governmental support from the Ministry of Health, Welfare and Sport.

Financial Disclosure:

All authors are (part-time or full) employees the Dutch Expertise Centre for Child Abuse.

Data sharing statement

The data, in Dutch, that support the findings of this study are available from the Dutch Expertise Centre for Child Abuse but restrictions apply to the availability of these data, and so are not publicly available. Data are, however, available from the authors upon reasonable request and with permission of DECCA.

For peer review only - http://bmjopen.bmj.com/site/about/guidelines.xhtml

References

1. Felitti VJ, Anda RF, Nordenberg D, et al. Relationship of childhood abuse and household dysfunction to many of the leading causes of death in adults. The Adverse Childhood Experiences (ACE) Study. *Am J PrevMed* 1998;14(4):245-58.
2. Gilbert R, Widom CS, Browne K, et al. Burden and consequences of child maltreatment in high-income countries. *Lancet* 2009;373(9657):68-81. doi: 10.1016/s0140-6736(08)61706-7 [published Online First: 2008/12/06]
3. Irazuzta JE, McJunkin JE, Danadian K, et al. Outcome and cost of child abuse. *Child Abuse Negl* 1997;21(8):751-57.
4. Gelles RJ, Perlman S. Estimated Annual Cost of Child Abuse and Neglect. Chicago IL: Prevent Child Abuse America,, 2012.
5. Royal Dutch Medical Association. KNMG-meldcode kindermishandeling en huiselijk geweld 2018 [Available from: https://www.knmg.nl/advies-richtlijnen/knmg-publicaties/meldcode-kindermishandeling-en-huiselijk-geweld-1.htm.
6. WHO Regional Office for Europe. European report on preventing child maltreatment. In: Sethi D, Bellis M, Hughes K, et al., eds., 2013.
7. Kindermishandeling LEC. [Available from: www.leck.nu accessed 5-10-2017.
8. European Parliament. 2018 reform of EU data protection rules 2018 [Available from: https://ec.europa.eu/commission/priorities/justice-and-fundamental-rights/data-protection/2018-reform-eu-data-protection-rules_en accessed 10-4-2019.
9. Brink FW, Thackeray JD, Bridge JA, et al. Child advocacy center multidisciplinary team decision and its association to child protective services outcomes. *Child Abuse Negl* 2015;46:174-81. doi: 10.1016/j.chiabu.2015.04.011 [published Online First: 2015/05/11]
10. Draus JM, Jr. A Multidisciplinary Child Protection Team Improves the Care of Nonaccidental Trauma Patients. *The American surgeon* 2017;83(5):477-81. [published Online First: 2017/05/26]
11. Wallace GH, Makoroff KL, Malott HA, et al. Hospital-based multidisciplinary teams can prevent unnecessary child abuse reports and out-of-home placements. *Child Abuse Negl* 2007;31(6):623-9. doi: 10.1016/j.chiabu.2006.12.010 [published Online First: 2007/06/26]
12. Otterman G, Jalsenius M, Maguire S, et al. Paediatric approaches to child maltreatment are subject to wide organisational variations across Europe. *Acta paediatrica (Oslo, Norway : 1992)* 2017;106(7):1110-17. doi: 10.1111/apa.13779 [published Online First: 2017/02/09]
13. Bechtel K, Ryan E, Gallagher D. Impact of sexual assault nurse examiners on the evaluation of sexual assault in a pediatric emergency department. *Pediatric emergency care* 2008;24(7):442-7. doi: 10.1097/PEC.0b013e31817de11d [published Online First: 2008/06/27]
14. Adams JA, Starling SP, Frasier LD, et al. Diagnostic accuracy in child sexual abuse medical evaluation: role of experience, training, and expert case review. *Child Abuse Negl* 2012;36(5):383-92. doi: 10.1016/j.chiabu.2012.01.004 [published Online First: 2012/05/29]

For peer review only - http://bmjopen.bmj.com/site/about/guidelines.xhtml

15. Hornor G, Thackeray J, Scribano P, et al. Pediatric sexual assault nurse examiner care: trace forensic evidence, ano-genital injury, and judicial outcomes. *Journal of forensic nursing* 2012;8(3):105-11. doi: 10.1111/j.1939-3938.2011.01131.x [published Online First: 2012/08/29]
16. McGuire L, Martin KD, Leventhal JM. Child abuse consultations initiated by child protective services: the role of expert opinions. *Academic pediatrics* 2011;11(6):467-73. doi: 10.1016/j.acap.2011.06.005 [published Online First: 2011/08/09]
17. Teeuw AH, Sieswerda-Hoogendoorn T, Aaftink D, et al. Assessments carried out by a child abuse and neglect team in an Amsterdam teaching hospital led to interventions in most of the reported cases. *Acta paediatrica (Oslo, Norway : 1992)* 2017;106(7):1118-27. doi: 10.1111/apa.13735 [published Online First: 2016/12/31]
18. Lindberg DM, Lindsell CJ, Shapiro RA. Variability in expert assessments of child physical abuse likelihood. *Pediatrics* 2008;121(4):e945-53. doi: 10.1542/peds.2007-2485 [published Online First: 2008/04/03]
19. Sege R, Flaherty E, Jones R, et al. To report or not to report: examination of the initial primary care management of suspicious childhood injuries. *Academic pediatrics* 2011;11(6):460-6. doi: 10.1016/j.acap.2011.08.005 [published Online First: 2011/10/15]
20. Talsma M, Bengtsson Bostrom K, Ostberg AL. Facing suspected child abuse--what keeps Swedish general practitioners from reporting to child protective services? *Scandinavian journal of primary health care* 2015;33(1):21-6. doi: 10.3109/02813432.2015.1001941 [published Online First: 2015/02/14]
21. Vulliamy AP, Sullivan R. Reporting child abuse: pediatricians' experiences with the child protection system. *Child Abuse Negl* 2000;24(11):1461-70. [published Online First: 2000/12/29]
22. Wegner SE, Humble CG, Feaganes J, et al. Estimated savings from paid telephone consultations between subspecialists and primary care physicians. *Pediatrics* 2008;122(6):e1136-40. doi: 10.1542/peds.2008-0432 [published Online First: 2008/12/03]

For peer review only - http://bmjopen.bmj.com/site/about/guidelines.xhtml

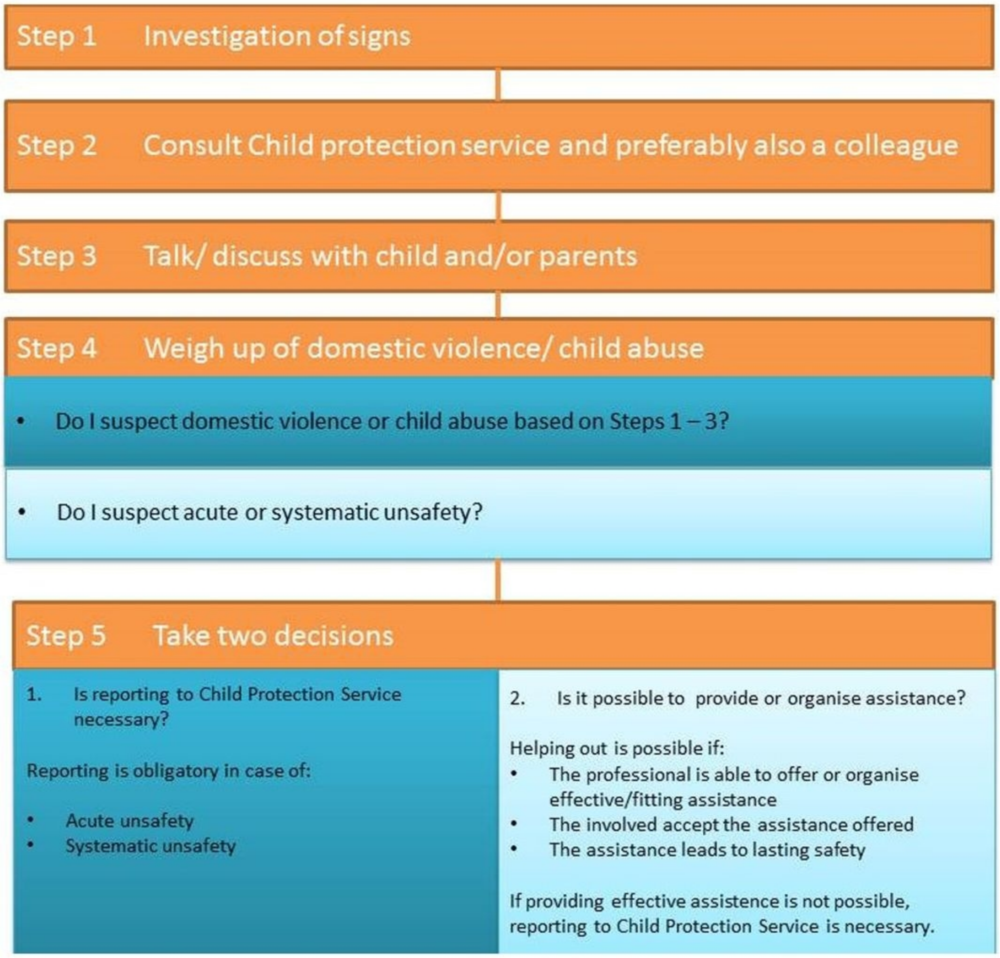

Figure 1. Stepwise approach of managing child abuse as guideline as published by the Royal Dutch Medical Association

90x90mm (300 x 300 DPI)

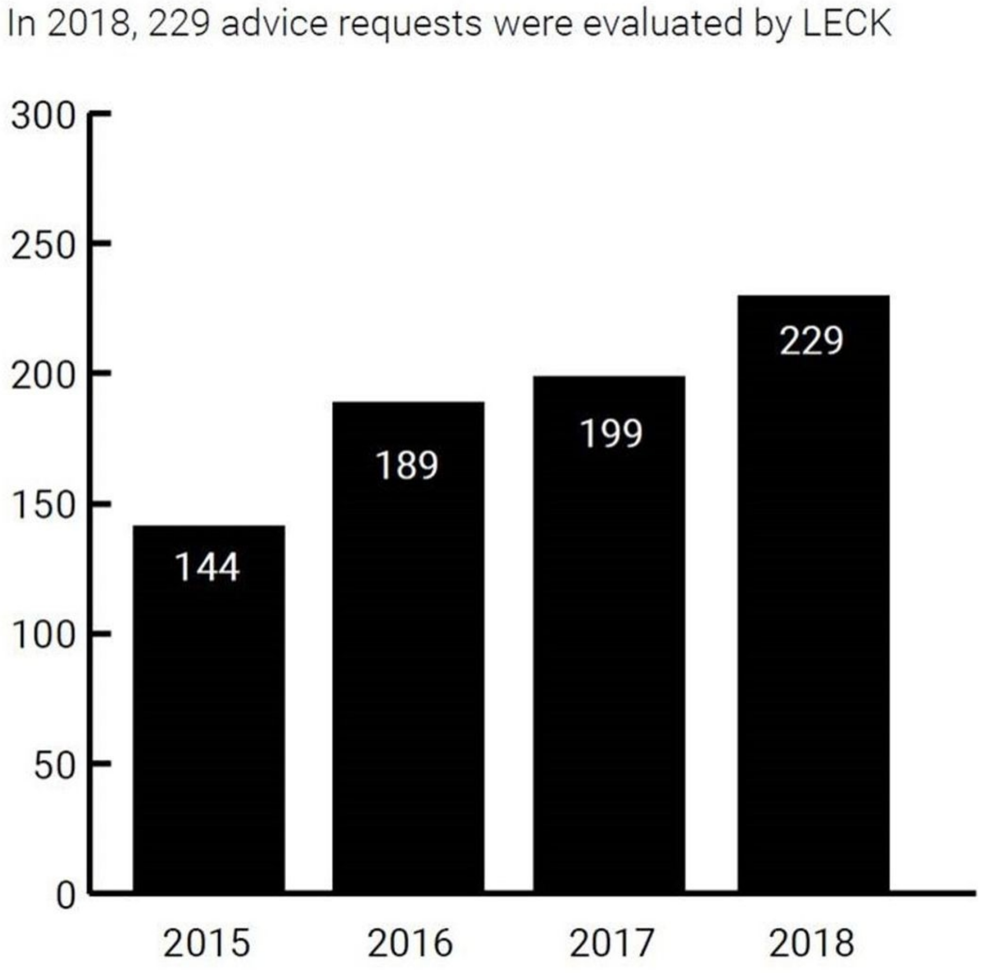

Figure 2.Number of DECCA advisory requests from 2015 to 2018.

90x90mm (300 x 300 DPI)

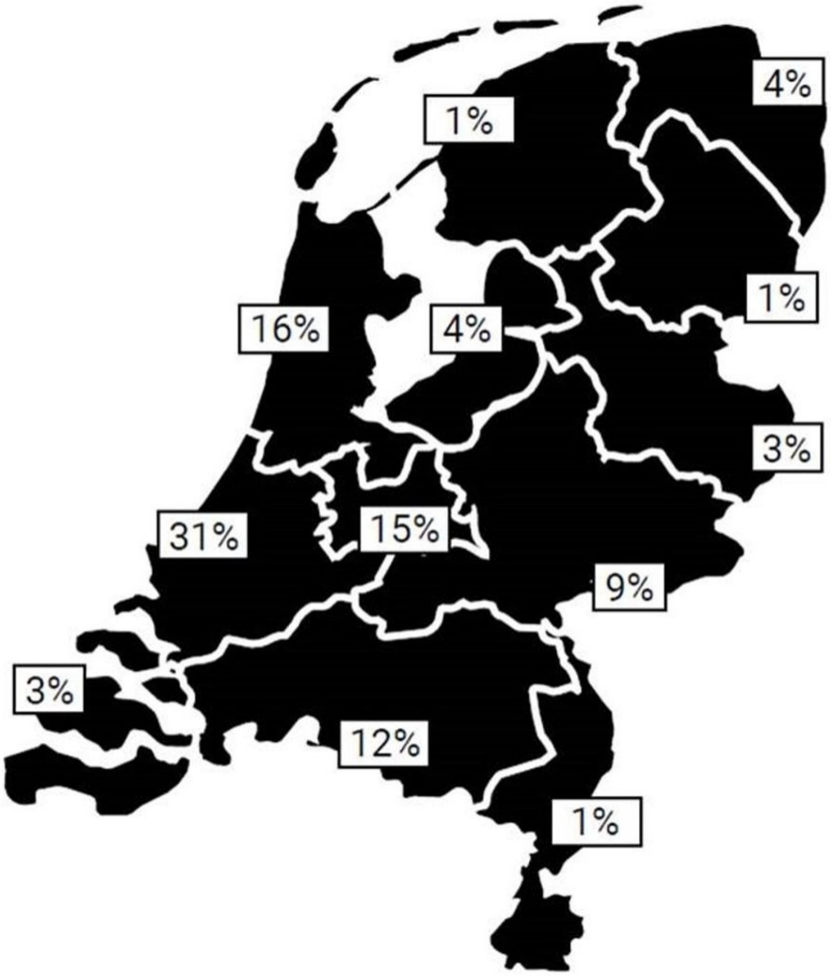

Figure 3. Geographic distribution of the advisee to the Dutch Expertise Centre for Child Abuse (N= 740, 21 missing).

90x90mm (300 x 300 DPI)

STROBE Statement - The start of the Dutch Expertise Centre for Child Abuse; a descriptive study of the implementation and data of the first 4 years

	Item	Recommendation	Page
**Title and abstract**	1	(*a*) Indicate the study's design with a commonly used term in the title or the abstract	Title page
		(*b*) Provide in the abstract an informative and balanced summary of what was done and what was found	3
**Introduction**			
Background/rationale	2	Explain the scientific background and rationale for the investigation being reported	6
Objectives	3	State specific objectives, including any prespecified hypotheses	7
**Methods**			
Study design	4	Present key elements of study design early in the paper	10
Setting	5	Describe the setting, locations, and relevant dates, including periods of recruitment, exposure, follow-up, and data collection	10
Participants	6	(*a*) *Cohort study*—Give the eligibility criteria, and the sources and methods of selection of participants. Describe methods of follow-up<br>*Case-control study*—Give the eligibility criteria, and the sources and methods of case ascertainment and control selection. Give the rationale for the choice of cases and controls<br>*Cross-sectional study*—Give the eligibility criteria, and the sources and methods of selection of participants	10
		(*b*) *Cohort study*—For matched studies, give matching criteria and number of exposed and unexposed<br>*Case-control study*—For matched studies, give matching criteria and the number of controls per case	N/A
Variables	7	Clearly define all outcomes, exposures, predictors, potential confounders, and effect modifiers. Give diagnostic criteria, if applicable	N/A
Data sources/ measurement	8	For each variable of interest, give sources of data and details of methods of assessment (measurement). Describe comparability of assessment methods if there is more than one group	N/A
Bias	9	Describe any efforts to address potential sources of bias	N/A
Study size	10	Explain how the study size was arrived at	N/A
Quantitative variables	11	Explain how quantitative variables were handled in the analyses. If applicable, describe which groupings were chosen and why	N/A
Statistical methods	12	(*a*) Describe all statistical methods, including those used to control for confounding	11
		(*b*) Describe any methods used to examine subgroups and interactions	N/A
		(*c*) Explain how missing data were addressed	N/A
		(*d*) *Cohort study*—If applicable, explain how loss to follow-up was addressed<br>*Case-control study*—If applicable, explain how matching of cases and controls was addressed	N/A

For peer review only - http://bmjopen.bmj.com/site/about/guidelines.xhtml

		Cross-sectional study—If applicable, describe analytical methods taking account of sampling strategy	
		(*e*) Describe any sensitivity analyses	N/A
Results			
Participants	13*	(a) Report numbers of individuals at each stage of study—eg numbers potentially eligible, examined for eligibility, confirmed eligible, included in the study, completing follow-up, and analysed	N/A
		(b) Give reasons for non-participation at each stage	N/A
		(c) Consider use of a flow diagram	N/A
Descriptive data	14*	(a) Give characteristics of study participants (eg demographic, clinical, social) and information on exposures and potential confounders	10
		(b) Indicate number of participants with missing data for each variable of interest	N/A
		(c) *Cohort study*—Summarise follow-up time (eg, average and total amount)	N/A
Outcome data	15*	*Cohort study*—Report numbers of outcome events or summary measures over time	N/A
		Case-control study—Report numbers in each exposure category, or summary measures of exposure	N/A
		Cross-sectional study—Report numbers of outcome events or summary measures	N/A
Main results	16	(*a*) Give unadjusted estimates and, if applicable, confounder-adjusted estimates and their precision (eg, 95% confidence interval). Make clear which confounders were adjusted for and why they were included	N/A
		(*b*) Report category boundaries when continuous variables were categorized	N/A
		(*c*) If relevant, consider translating estimates of relative risk into absolute risk for a meaningful time period	N/A
Other analyses	17	Report other analyses done—eg analyses of subgroups and interactions, and sensitivity analyses	N/A
**Discussion**			
Key results	18	Summarise key results with reference to study objectives	13
Limitations	19	Discuss limitations of the study, taking into account sources of potential bias or imprecision. Discuss both direction and magnitude of any potential bias	15
Interpretation	20	Give a cautious overall interpretation of results considering objectives, limitations, multiplicity of analyses, results from similar studies, and other relevant evidence	13
Generalisability	21	Discuss the generalisability (external validity) of the study results	16
**Other information**			
Funding	22	Give the source of funding and the role of the funders for the present study and, if applicable, for the original study on which the present article is based	16

For peer review only

For peer review only - http://bmjopen.bmj.com/site/about/guidelines.xhtml
